# Melittin alcalase-hydrolysate: a novel chemically characterized multifunctional bioagent; antibacterial, anti-biofilm and anticancer

**DOI:** 10.3389/fmicb.2024.1419917

**Published:** 2024-07-17

**Authors:** Samia E. El-Didamony, Mohamed H. Kalaba, Mohamed H. Sharaf, Esmail M. El-Fakharany, Ali Osman, Mahmoud Sitohy, Basel Sitohy

**Affiliations:** ^1^Department of Zoology and Entomology, Faculty of Science, Al-Azhar University (Girls), Nasr City, Egypt; ^2^Department of Botany and Microbiology, Faculty of Science, Al-Azhar University (Boys), Cairo, Egypt; ^3^Protein Research Department, Genetic Engineering and Biotechnology Research Institute (GEBRI), City of Scientific Research and Technological Applications (SRTA-City), New Borg Al-Arab City, Alexandria, Egypt; ^4^Pharmaceutical and Fermentation Industries Development Center, City of Scientific Research and Technological Applications (SRTA-City), New Borg Al-Arab City, Alexandria, Egypt; ^5^Pharos University in Alexandria, Alexandria, Egypt; ^6^Department of Biochemistry, Faculty of Agriculture, Zagazig University, Zagazig, Egypt; ^7^Department of Clinical Microbiology, Infection, and Immunology, Umeå University, Umeå, Sweden; ^8^Department of Diagnostics and Intervention, Oncology, Umeå University, Umeå, Sweden

**Keywords:** bee venom, melittin, *Apis mellifera*, antibacterial activity, anticancer activity, enzymatic hydrolysis, electro-spray ionization, wound healing assay

## Abstract

The prevalent life-threatening microbial and cancer diseases and lack of effective pharmaceutical therapies created the need for new molecules with antimicrobial and anticancer potential. Bee venom (BV) was collected from honeybee workers, and melittin (NM) was extracted from BV and analyzed by urea-polyacrylamide gel electrophoresis (urea-PAGE). The isolated melittin was hydrolyzed with alcalase into new bioactive peptides and evaluated for their antimicrobial and anticancer activity. Gel filtration chromatography fractionated melittin hydrolysate (HM) into three significant fractions (F1, F2, and F3), that were characterized by electrospray ionization mass spectrometry (ESI-MS) and evaluated for their antimicrobial, anti-biofilm, antitumor, and anti-migration activities. All the tested peptides showed antimicrobial and anti-biofilm activities against Gram-positive and Gram-negative bacteria. Melittin and its fractions significantly inhibited the proliferation of two types of cancer cells (Huh-7 and HCT 116). Yet, melittin and its fractions did not affect the viability of normal human lung Wi-38 cells. The IC_50_ and selectivity index data evidenced the superiority of melittin peptide fractions over intact melittin. Melittin enzymatic hydrolysate is a promising novel product with high potential as an antibacterial and anticancer agent.

## Introduction

Infectious diseases are considered one of the significant causes of death worldwide (Waldman and Sato, 2016). Although many antimicrobial agents have been developed, the misuse of these treatments generated severe resistance among several microorganisms, manifested as multi-drug resistant pathogens (MDR) (Morens and Fauci, 2012). MDR pathogens are typically related to nosocomial bacterial infections, have become a common cause of bacterial community-acquired illnesses, and are responsible for human morbidity and mortality nowadays (van Duin and Paterson, 2020). The risk of death from resistant strains is twice as high as that of non-resistant ones, causing a stressful impact on the medical and public community (Khabbaz et al., 2014).

Also, Cancer is one of the deadliest and the most aggressive diseases in the world, and is ranked as the second leading cause of death (Ritchie et al., 2018; Azevedo et al., 2020). In 2020, new cancer cases were estimated to be 19.3 million, and almost 10.0 million cancer deaths occurred. Predictably, the growth and elderly population may increase the number of new cancer cases to 28.4 million in 2040, with about a 47% rise from 2020 (Ferlay et al., 2021). Despite employing many treatment modalities, e.g., surgery, chemotherapy, radiotherapy, anti-angiogenic therapy, and laser treatment, have been employed, no complete success has been achieved due to the insufficient effect of these therapies and their severe side effects (Wilson et al., 2015; Mansoori et al., 2017; Sitohy et al., 2017).

Therefore, it is critical to discover and produce innovative and non-traditional treatments to combat these diseases. Changes in the expression of some peptides was found to be implicated in the pathogenesis and treatment of colon cancer (Sitohy and El-Salhy, 2001, 2002, 2003; El-Salhy and Sitohy, 2002; El-Salhy et al., 2003; Rashad et al., 2018; AbdelMageed et al., 2019, 2021; Ali et al., 2019, 2021; Olsson et al., 2020; Eltorky et al., 2024)en used for several years in folk medicine. One of the natural medicines is bee venom therapy (Gupta and Stangaciu, 2014). Bee venom (BV) is an api-toxin secreted by the bee honeybees (*Apis mellifera*). BV is composed of complex mixture peptides such as melittin, apamine, adolapin, and MCD peptide, enzymes like phospholipase A2 (PLA2), hyaluronidase, acid phosphomonoesterase, and lysophosphofolipase, and also containing various amines, such as histamine, dopamine, and norepinephrine (Son et al., 2007).

A series of recent studies indicated that BV has a wide range of microbial activity against bacteria, fungi, and viruses (El-Didamony et al., 2022b; Pérez-Delgado et al., 2023), as well as treating different types of cancer cells (Taher et al., 2017; El-Didamony et al., 2022a). Overall, BV and its selective components are considered promising agents for disease management. In addition, BV decreases the adverse effects of other types of medications and conventional drugs (Hassan et al., 2021). However, the efficacy of BV appears to be because of melittin; therefore, this peptide might be a better choice than BV in its native form (Rady et al., 2017). Melittin (C_131_H_229_N_39_O_31_) is the main active component of BV, representing more than 50 % of its total dry weight. It is a cationic, lytic peptide constituted by 26 amino acid residues. The first 20 residues (N-terminal) of the structure of melittin are predominantly hydrophobic amino acids, whereas the carboxyl-terminal of the peptide is mainly composed of hydrophilic residues (Vogel and Jähnig, 1986). Melittin is an amphoteric molecule in which the carboxy-terminal region is hydrophilic. In contrast, the amino-terminal region is hydrophobic due to a series of positively charged amino acids and reported to have strong hemolytic activity (Renzo et al., 1988). Based on cationic property of melittin, it exhibited strong antimicrobial effects through interaction with bacterial membranes depending on their physiochemical properties such as helicity, length, and net charge (Geitani et al., 2019). The positive charge can enable proteins or peptides to bind to DNA (Sitohy et al., 2001b,^2002^), and this affects the biological replication process (Sitohy et al., 2001a,^2006^). There is a plethora of research evidencing the antibacterial action of cationic proteins and peptides (Mahgoub et al., 2011; Sitohy et al., 2012; Abdel-Shafi et al., 2016, 2019a,b; Osman et al., 2016a). Melittin also displayed a significantly broad spectrum of antitumor and cytotoxic effects on multiple types of cancer (Zhang and Sun, 2024). Hence, this work aimed to isolate the melittin as main active component of BV to be the substrate for further amelioration by alcalase hydrolysis. Alcalase hydrolysis of native proteins was reported to generate bioactive and antibacterial peptides (Osman et al., 2021a). Several research works proved that enzymatic hydrolysis generates highly bioactive peptides from protein sources (El-Zahar et al., 2003; El-Zahar et al., 2004; Rayaprolu et al., 2015; Abdel-Hamid et al., 2016, 2017; Osman et al., 2016b,2021b; Al-Mohammadi et al., 2020). The generated peptides can elicit bioactivity, including anti-proliferative action against cancer cell lines. Studies have shown that enzyme catalysis provides peptides from various food sources that can act as physiological biomolecules (Rayaprolu et al., 2017). Nevertheless, no previous references demonstrated the biological impact of enzymatic hydrolysis on melittin. Therefore, the current study aimed to develop new bioactive peptides through alcalase enzymatic hydrolysis of melittin and evaluate their antibacterial and anticancer activity against multidrug-resistant (MDR) gram-negative and gram-positive bacteria. The study also determined cytotoxicity and anticancer activity of melittin on normal and cancer cell respectively.

## Materials and methods

### Bee venom collection

Bee venom (BV) was collected from healthy workers of the honeybee, *Apis mellifera* (L.) according to Fakhim-Zadeh (1998) and Walaa et al. (2017) using the electro-stimulation method. The electric shock device (VC-6F model from Apitronic Services, 9611, No. 4 Road, Richmond, B.C., Canada) comprises a frame with wire electrodes installed in parallel to each other. The frame was mounted on the top or under the hive before connecting to an electro-stimulator. The electrical impulses stimulated the bee workers to sting through latex, placed on a glass plate. The glass plate was carefully transferred to the laboratory, and the venom was dried at ambient temperature. A sharp scraper was used to scrape off the dry venom. Then, fresh bee venom was stored in dark glass tubes at a temperature of −4°C until needed. A stock solution of the venom was prepared and centrifugated at 8000 rpm, for 5 min. The supernatant was lyophilized and kept at –80°C until use.

### Characterization of bee venom using Native-PAGE

The lyophilized bee venom was dissolved in a buffer solution at a concentration of 5 mg/ml and a pH of 6.8. The buffer contained 0.25 M Tris base and 50% glycerol. The analysis was conducted using native polyacrylamide gel electrophoresis (native-PAGE) following the method described by Laemmli (1970). Stacking and resolving gels with 3% and 8% acrylamide concentrations, respectively, were used for the analysis. The electrode buffer used in the experiment had a pH of 8.3 and consisted of a solution containing 0.125 M Tris base and 0.96 M glycine.

### Melittin isolation

Melittin was precipitated from BV by the addition of two volumes of saturated picric acid. Melittin-picrate was collected by centrifugation at 10,000 g for 15 min at 4°C, washed twice with 70% saturated picric acid, and finally cleaved by adding 2 ml acetone containing 0.2% concentrated HCl. After three washes with the latter solvent, melittin was recovered as a slightly brown powder. Traces of picric acid could be removed by passing an aqueous solution of melittin, at pH about 3, over Dowex-1 in the chloride form (Kreil, 1973).

### Characterization of isolated melittin using Urea-PAGE

Lyophilized bee venom, melittin and lysozyme were dispersed (10 mg/ml) in pH 6.8 buffer containing 0.25 M Tris-base, 50% glycerol and bromophenol blue traces. Samples were centrifuged at 15,000 × g for 5 min at 20°C. Supernatants were analyzed by urea-PAGE (10 μL of protein/lane) in 3% and 12% stacking and resolving gels, respectively (Evans and Williams, 1980).

### Hydrolysis of melittin, fractionation, and characterization

#### Melittin alcalase hydrolysis

Melittin was hydrolyzed by alcalase at 1:200 w/w enzyme/substrate ratio at 50°C in 0.1 M Na_2_HPO_4_–NaH_2_PO_4_ buffer at pH 7 for 2h. At the end of the hydrolysis period, the enzyme was inactivated by placing the reaction mixture in a boiling water bath for 10 min. The hydrolysate was centrifuged at 4000 × g for 15 min and the supernatant was lyophilized then stored at −20°C (Osman et al., 2016b). Trinitrobenzene sulfonic acid (TNBS) technique (Adler-Nissen, 1979) determined the degree of hydrolysis (DH), measuring the absorbance at 340 nm using a UV/Visible spectrophotometer while using L-leucine as the standard.

#### Melittin hydrolysate fractionation

The lyophilized melittin hydrolysate (100 mg) was suspended in 10 ml of deionized water before being separated using a Sephadex G-25 gel filtration column (1.6 x 150 cm). At a flow rate of 1 ml/min, two milliliters were injected, eluted with pure water, and detected at 280 nm. For assessing their antibacterial activity, the main peaks were collected and lyophilized.

#### Electro-Spray-Ionization-Mass-Spectrometry (ESI-MS)

After the melittin sample was broken down for two hours with alcalase, it was analyzed using electrospray ionization mass spectrometry (ESI-MS) in both positive and negative ion modes. The parameters were as follows, vaporizer temperature at 320^°^C, heated capillary serving simultaneously as repeller electrode (20V) at 180^°^C, corona voltage 4 kV, electro multiplier voltage 1.6 kV. Nitrogen was used as both sheath (50 psi) and auxiliary gas, and argon as collision gas at a pressure of 1.9 mTorr. The mass spectrometer was operated in full ion scanning, which was chosen to detect positive and negative ions, for total scan durations of 1.05. The offset voltage was −15V. The ions represented the protonated molecular ion [M+H+], [M+K+], [M+Na+] and [M-H+] for the polypeptide in hydrolysate. The masses of the resulting peptides were matched to the original amino acid sequence of melittin registered in https://www.uniprot.org/.

#### Antibacterial activity and minimum inhibitory concentration (MIC) determination

The antibacterial activity of melittin, melittin hydrolysate (HM) and its peptide fractions (F1, F2, and F3) were evaluated on Muller Hinton agar (MHA, India) using agar diffusion assay. An overnight culture of *Staphylococcus aureus* ATCC 6538, *Bacillus cereus* ATCC 10987, *Enterococcus faecalis* ATCC29212 *Escherichia coli* ATCC 8739, *Salmonella typhimurium* ATCC14028 and *Pseudomonas aeruginosa* ATCC 9072 were lawned on the surface of prepared MHA. Agar wells (6 mm) were cut using a sterile corkborer then 100 μl of each compound was transferred to the well and left for 2 h at 4°C followed by incubation for 24 h at 37°C. In addition, Rifampin was used as antibiotic control. After incubation, the inhibition zones were measured and recorded and this experiment was repeated three times (Wayne, 2015; Kalaba et al., 2022). MIC values of melittin, melittin hydrolysate (HM) and its fractionation (F1, F2, and F3) were performed at a Concentration (from 10 mg/ml to 1.21 μg/ml) against all bacterial strains (Khalifa et al., 2013). A 100 μl test sample of different concentrations was added to sterile microtiter plate wells filled with 100 μl of double-strength Mueller Hinton (MH) broth. Bacterial cell suspension (50μl) with OD comparable to 0.5 McFarland standard was applied to all wells containing the tested compounds. Positive control wells were filled with MH broth and bacterial suspension to ensure that the broth was enough to sustain bacterial growth. The negative control wells contained sterile distilled water and Mueller Hinton broth to ensure sterility. The plates were incubated at 37°C for 24 hours and the experiment was repeated three times. To detect bacterial growth, 30 μl of resazurin solution (0.02% wt/v) (HiMedia) was add to each well and the plate was re-incubated overnight. A shift in color from blue to purple, red, or pink indicated bacterial growth, while no change in color suggested that there were no growth present (El-Sherbiny et al., 2022).

#### Evaluation of the antibiofilm activity

A 96-well flat bottom microtiter plate (Merck, Germany) was used to determine the anti-biofilm activity of the melittin, melittin hydrolysate (HM) and its fractionation (F1, F2, and F3) against gram-positive (*Staphylococcus aureus*) and gram-negative (*Pseudomonas aeruginosa*) as described by El-Didamony et al. (2022b) with some modifications. The wells of the plates were filled with 100 μl of Mueller Hinton (MH) broth medium. Subsequently, 100 μl of the tested compounds at concentrations started from MIC were added and subjected to double-fold dilution to reach 1/8 MIC, then thoroughly mixed. All wells were inoculated with the tested bacteria and incubated statically for 24 h at 37°C. In addition, positive control was performed, containing the tested bacteria inoculated in MH broth only. After incubation, the contents of the wells of the microtiter plates were discarded and gently washed with phosphate-buffered saline (PBS, pH 7.2) to remove free-floating non-adherent cells from the walls and bottom of the wells. The wells of the microtiter plates were then air-dried for 45 min. After drying, adherent “sessile” *S. aureus*, and *P. aeruginosa* in the wells were fixed with absolute alcohol, and the wells were then flooded with crystal violet stain (0.1%, w/v) and incubated in the dark for 30 min. Afterward, the wells were thoroughly washed with sterile deionized water until all excess dye was removed. The plates were then air-dried again. After complete drying, 200 ml of 33% acetic acid was added to each well, absorbance at 630 nm was measured, and the percentage of inhibition of biofilm formation was calculated using the following equation:


Biofilm inhibition % = (1-(OD of treated cells / OD of control cells)) × 100

### Cytotoxicity and antitumor assay

#### Cell culture

Human liver carcinoma (Huh-7) cells and colon carcinoma (HCT 166) cells were used as cancer cell lines, while human lung fibroblast (Wi-38) cells have been used as normal cell lines in this search. Both cells were obtained from American Type Culture Collection (ATCC, USA). Normal and cancer cells were cultured in DMEM (Lonza, USA) supplemented with 10% fetal bovine serum (Gibco, USA) and cell lines were maintained at 37°C and 5% CO_2_.

#### MTT assay

As described by El-Didamony et al. (2022a), both cancer and normal cell lines were cultured in 96 well tissue culture microtiter plates (Nunc-Denmark) at a concentration of 1 X 10^5^ cells/ml (100 μl / well) with RPMI-164 growth medium (Gibco BRL, Grand Island, NY) and supplemented with 10% fetal bovine serum (FBS), 1mM sodium pyruvate, 2mM L-glutamine and antibiotics (penicillin 100 IU/ml, streptomycin 100μg/ml) at 37°C and 5% CO_2_ for 24 h to develop a complete monolayer sheet. After a confluent sheet of cells was formed, the growth media was decanted, and the cells were treated with the test materials. Cell viability was evaluated using the 3-[4, 5-dimethylthiazol]-2, 5-diphenyltetrazolium bromide method (MTT assay) to determine the activity of the melittin and its peptide derivatives (1-4 peptides) against both cancer and normal cells lines according to methods of Mosmann (1983) and Dawoud et al. (2022). Normal and cancer cells (1 × 10^4^) sterile 96-well microplates were incubated, after which the formed monolayer sheet of cells was separated, and the growth media was decanted. Cells were treated with melittin, melittin hydrolysate (HM) and its fractionation parts (F1, F2, and F3) at different concentrations of 25, 50, 100, 200, 400 and 800 μg/ml in the volume of 100 μl / well and the control was added to saline of equal volume. All plates were incubated for another 72 h in 5% CO_2_ incubator. Afterward, the media was removed, and the cells were washed with phosphate-buffered saline (PBS). The plates were incubated with 50μl/well of MTT solution (Sigma -Aldrich, 0.5 mg/ml) for 4-5 h, then 200 μl / well of DMSO solution was added. Finally, the absorbance of each well was measured at 590 nm wavelength using an ELIZA reader (BMG LabTech, Germany). The test was done in triplicates to all cells. The viability percent was calculated as follows:


Viability%=(Mean⁢OD⁢Treated)/(Mean⁢OD⁢Control)×100

Where OD is optical density.

The anticancer activity of the melittin and its peptide derivatives was estimated by calculating the IC_50_ values. The IC_50_ is the concentration of tested material required to inhibit 50% of cell growth. Also, the value of the selectivity index (SI) that indicates the ratio of the IC_50_ value of normal cells versus the IC_50_ value of cancer cells was included as reported by Abu-Serie and El-Fakharany (2017) and Uversky et al. (2017).

#### Morphological analysis

The effect of melittin, melittin hydrolysate (HM) and its fractionation (F1, F2, and F3) on cell morphology was observed on HCT 166 cells as a model among the tested cell lines. Cells were seeded in 12-well plates containing DMEM (Lonza, USA) supplemented with 10% fetal bovine serum (Gibco, USA) at a density of 5 × 10^5^ cells/well and incubated for 24 h. Then the media were removed and treated with (100, 200 and 400 μg/ml) of the melittin and its peptide derivatives. Morphological changes in cells were observed using phase-contrast microscopy (Olympus, Germany) as compared to untreated cells.

#### Cell migration inhibition assay

To estimate the anti-migration potency of melittin and its peptide derivatives, the wound healing test was performed according to Han et al. (2018). Huh-7 cell line was selected for this assay; we seeded the cells at a density of 1 × 10^5^ cells/well in 12-well plates. When the cells became around 90% confluence, cells were scratched with a sterile micropipette tip to make a wound down through the confluent cell monolayer. Then, we washed the cells with PBS and cultured in FBS-free DMEM with IC_50_ concentration of melittin and its peptide derivatives. After that, the wound closure area was determined and recorded by imaging analysis cellSens software (Olympus, Japan) at 0, 48 and 72 h. The inhibition in closure area was calculated to estimate the migration percentages in the treated wells versus untreated wells as follows.


Inhibition⁢migration%=

[100-(scratchwidthat0h-scratchwidthatobservationtime)/

scratchwidthat0h×100%].

### Statistical analysis

All tests were performed in three replicates and the data were represented as Mean ± standard error (SE) or mean ± standard deviation (SD) using Sigmaplot 12.5 and Microsoft office 365. IC_50_ value was calculated using Graph Pad Prism 6.0 software.

## Results

### Characterization of bee venom and isolated melittin

**FIGURE 1:**
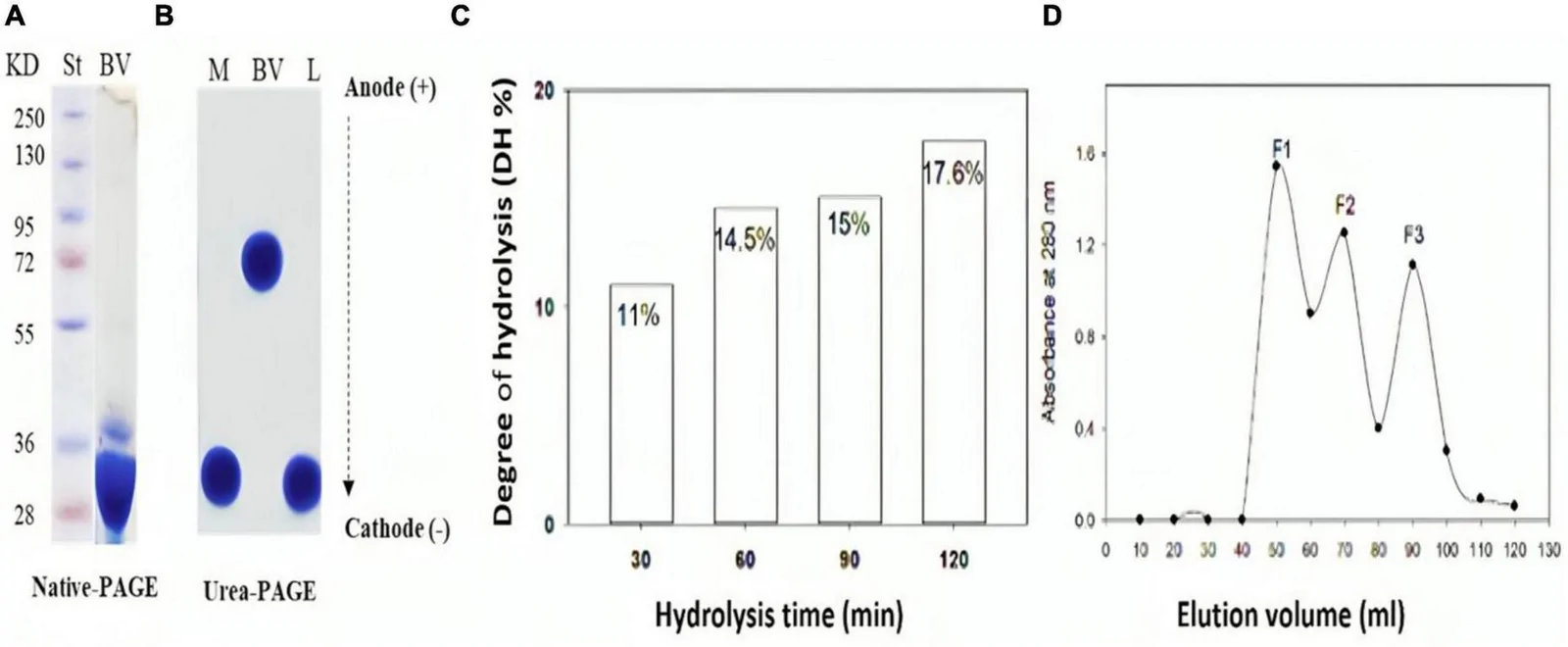
Chemical characterization of bee venom (BV) and its main component (Melittin) before and after hydrolysis with alcalase (E/S ratio 1:200) for 120 min. **(A)** SDS-PAGE of BV, **(B)** Urea-PAGE of BV, melittin and lysozyme **(C)** Degree of hydrolysis (DH %) of melittin in 2 hours alcalse treatment **(D)** Sephadex G-25 Gel filtration chromatography of melittin hydrolysate obtained by 60 min-alcalase hydrolysis with 15% DH. L; lysozyme in **(B).**

Native polyacrylamide electropherogram of bee venom (BV), presented in Figure 1A, indicates two major bands of around 28 and 36 kDa. The melittin (M), a 26-amino acid polypeptide, was extracted from bee venom and analyzed by urea-polyacrylamide gel electrophoresis (urea-PAGE), as compared to bee venom and lysozyme (L) as a basic protein control (Figure 1B). The migration of urea-PAGE towards the cathode direction demonstrated that M and L exhibited significantly higher migration rates compared to the native bee venom protein (BV), indicating a large positive charge on M comparable to L.

Figure 1C shows the degree of hydrolysis of melittin after exposure to alcalase at an E/S ratio of 1:200 for 120 minutes. Enzymatic degradation steadily gets greater over time. Variations in DH are typically caused by changes in the enzyme reaction time, which affects peptide bond breakage. Maximum hydrolysis degree (DH 17.6 %) occurred after 2 h alcalase hydrolysis.

Melittin hydrolysates with varying degrees of hydrolysis were tested for their antibacterial efficacy against G+ and G- bacteria. Amazingly, 60 min-melittin hydrolysate (DH of 15%) uniquely exhibited potent antibacterial activity against the tested microorganisms. Consequently, this hydrolysate was chosen for further detailed research. This hydrolysate was further fractionated by, giving out three significant absorbance peaks (F1, F2, and F3) at 226 nm, (Figure 1D). Each peak’s related fractions were gathered, lyophilized to concentrate them, and then tested for different activities.

### Electro-spray-ionization-MS (ESI-M)

**FIGURE 2:**
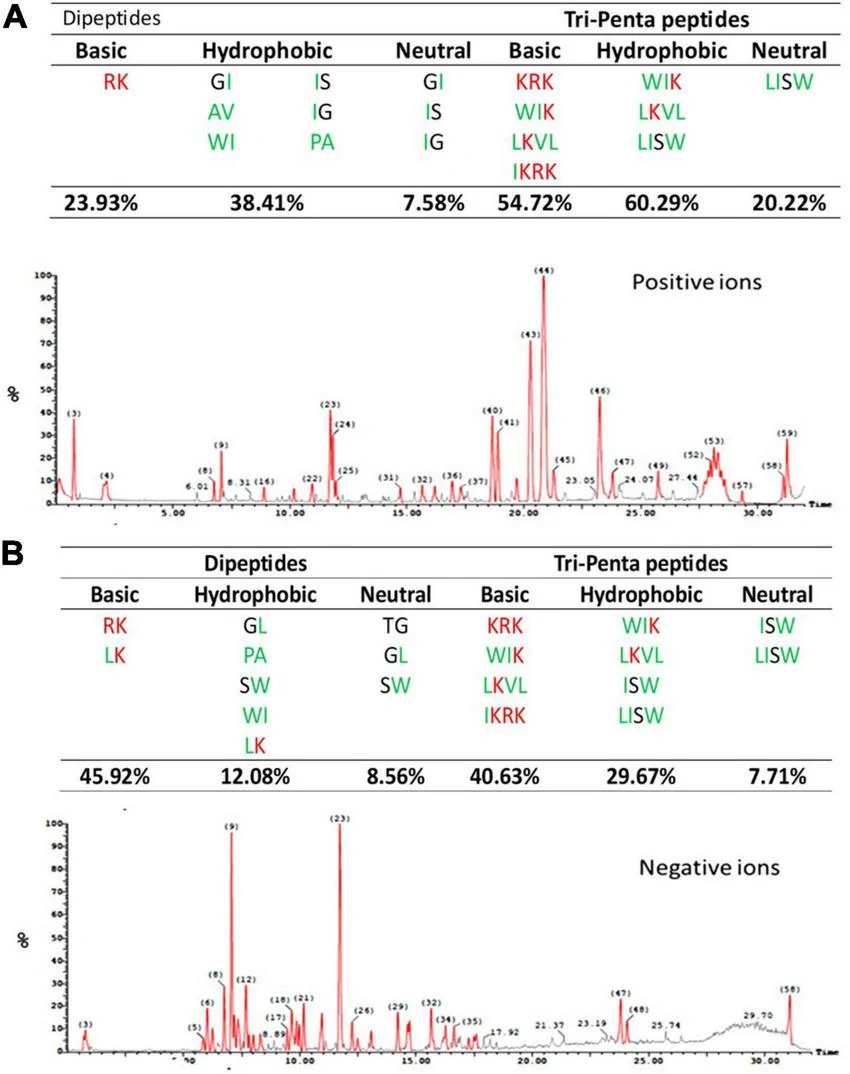
Chromatogram of peptides released from melittin by alcalase hydrolysis for one hour (15% DH) including positive and negative ions by electro-spray-ionization-MS (ESI-MS) in the positive **(A)** and negative **(B)** mode. The amino acid sequence of melittin is (GIGAVLKVLTTGLPALISWIKRKRQQ) according to Guha et al. (2021).

The ESI-MS chromatogram and data of the melittin hydrolysate produced by alcalase hydrolysis (15% DH) for one hour at optimum conditions are shown in Figure 2. It can be seen that basic and hydrophobic peptides represented the majority of the total peptides either as dipeptides or tri-penta peptides. The basic dipeptides amounted to 23.9 and 45.9 of the total dipeptides in the ESI-MS positive and negative mode, while the basic tri-penta-peptides represented 54.7 and 40.6, respectively. The hydrophobic dipeptides accounted for 38.4 and 12.08% of the total dipeptide in the positive and negative ESI-MS mode, respectively against 60.3 and 29.7% for the hydrophobic tri-penta peptides. Acidic peptides were absent from the melittin hydrolysate, while the neutral peptides were at minimal levels. One dipeptide (LK), one tripeptide (WIK) and one tetrapeptide. Moreover, three dipeptides (MK, LK, and WK), one tripeptide (WIK), and one tetrapeptide (LKVL) were simultaneously of basic and hydrophobic nature.

### Antimicrobial activity and MIC determination

The results of antimicrobial testing for melittin, melittin hydrolysate (HM), and its peptide fractions F1, F2, and F3 demonstrate broad-spectrum efficacy against tested bacteria, with inhibition zone diameters ranging from 13.3 to 48.6 mm, as shown in Table 1 and Supplementary Figure 1. The findings suggest that the examined compounds had varied levels of antibacterial activity, hydrolysate melittin (HM) and fraction F1 frequently having more effective effects across many strains than the native melittin (NM) and its fractions F2, and F3. Antibiotic controls had various results: Rifampin’s inhibition zone diameter ranged from 11.3 to 22 mm against the tested microorganisms. Considering the substances under investigation, it was observed that NM and its fractions F1, F2, and F3 consistently displayed greater minimum inhibitory concentrations (MICs) than HM. This tendency was observed in numerous bacterial strains. Table 2 shows the MIC values for the tested compounds, which varied from 2.44 μg/ml to 10000 μg/ml. Notably, these compounds are more effective against gram-positive bacteria than gram-negative bacteria in general. Specifically, compared to *Pseudomonas aeruginosa* with MIC at 10 mg/ml, *Staphylococcus aureus* demonstrated a lower MIC value toward HM at 2.44 μg/ml. Comparing the inhibition zones of native melittin (NM), alcalase hydrolyzed melittin (HM), and its fractions (F1, F2, and F3) against different bacterial strains provides valuable insights into their effectiveness.

**TABLE 1:** Antibacterial activity of native melittin (NM), alcalase hydrolysate melittin (HM) and its three main fractions (F1, F2, and F3), as compared to a control antibiotic (RA), after 24 h of incubation at 37°C.

Bacterial strain	Mean of inhibition zone diameter (mm) ± standard error values					
	NM	HM	F1	F2	F3	RA
*E. faecalis*	45 ± 0.5	48.6 ± 0.6	44 ± 0.5	45.3 ± 0.8	46.6 ± 0.8	21 ± 0.5
*S. aureus*	41 ± 0.5	41 ± 0.5	44.6 ± 0.8	36 ± 0.5	42 ± 0.5	25 ± 0.5
*S. typhimurium*	36.3 ± 0.8	38 ± 0.5	38.3 ± 0.3	36.6 ± 0.8	36 ± 0.5	11.3 ± 0.3
*E. coli*	34 ± 0.5	36.3 ± 0.6	38 ± 0.5	36.3 ± 0.3	35.3 ± 0.3	15.3 ± 0.3
*B. cereus*	27 ± 1.1	30 ± 0.5	32.3 ± 1.7	28 ± 0.5	29.3 ± 0.8	21.6 ± 0.6
*P. aeruginosa*	16 ± 0.5	13.3 ± 0.3	14.3 ± 0.3	18.3 ± 0.3	19.3 ± 0.3	11.3 ± 0.3

**TABLE 2:** Minimum inhibitory concentration (MIC) of native melittin (NM), hydrolysate melittin hydrolysate (HM) and its fractions (F1, F2, and F3).

Bacterial strain	MIC (μg/ml)				
	NM	HM	F1	F2	F3
*E. faecalis*	312	156	156	625	312
*S. aureus*	9.8	2.4	4.9	9.8	4.9
*S. typhi*	312	156	156	1250	312
*E. coli*	1250	625	625	5,000	1250
*B. cereus*	10,000	5,000	5,000	10,000	2,500
*P. aeruginosa*	10,000	10,000	10,000	10,000	10,000

### Anti-biofilm activity

**FIGURE 3:**
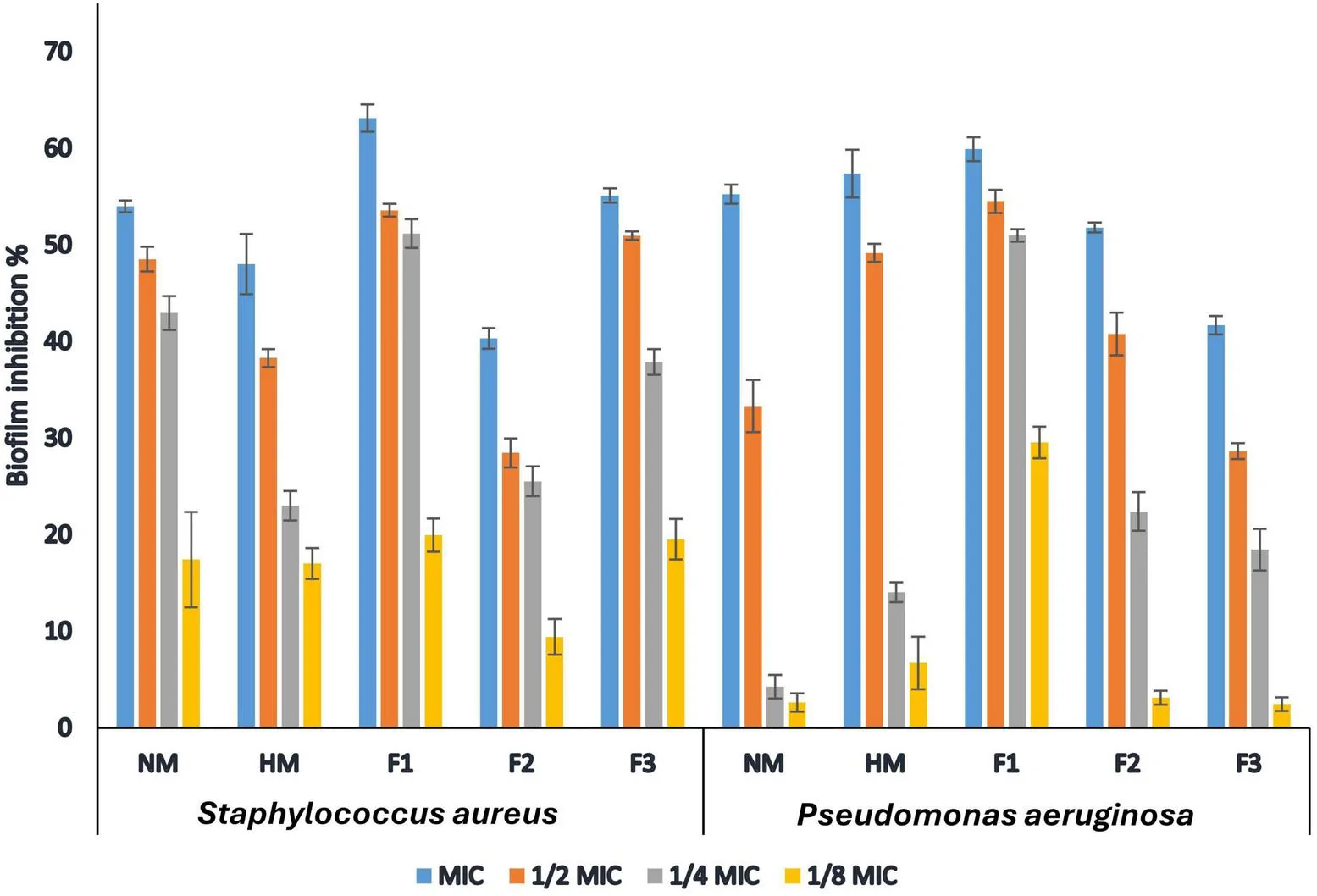
Inhibitory action of native melittin (NM), hydrolysate melittin (HM) and its peptide fractions (F1, F2 and F3) against biofilm formation by *S. aureus* and *P. aeruginosa*.

The current study evaluated the in *vitro* anti-biofilm activity of melittin, HM and F1, F2, and F3 against the biofilm-forming *S. aureus* and *P. aeruginosa.* The bacteria were grown in 96-well microtiter plates treated at different concentrations (MIC,1/2MIC,1/4MIC,1/8MIC for each strain separately) for 24 h. The results showed that all tested compounds had a concentration-dependent inhibitory effect against the tested strains, where fraction F1 exhibited the highest activity against biofilm produced by *S. aureus* with a 63.12% inhibition rate at 1.0 MIC concentration. Melittin, HM, and fractions F2 & F3 also showed varying degrees of effectiveness in inhibiting biofilm formation by *S. aureus*, with inhibition rates ranging from 40.32% to 55.11% at MIC concentration, respectively. Similarly, fraction F1 displayed the highest activity against biofilm formation by *P. aeruginosa* with a 59.93% inhibition rate, while melittin, HM, and fractions F2 & F3 exhibited antibiofilm activity with inhibition rates ranging from 41.7% to 57.39% at 1.0 MIC concentration as shown in Figure 3. In this study, fraction F1 exhibited the highest activity against biofilm formation by both *S. aureus* and *P. aeruginosa*, showing a 63.12% and 59.93% inhibition rate at 1.0 MIC concentration, respectively.

#### Anticancer and cytotoxicity assay

**FIGURE 4:**
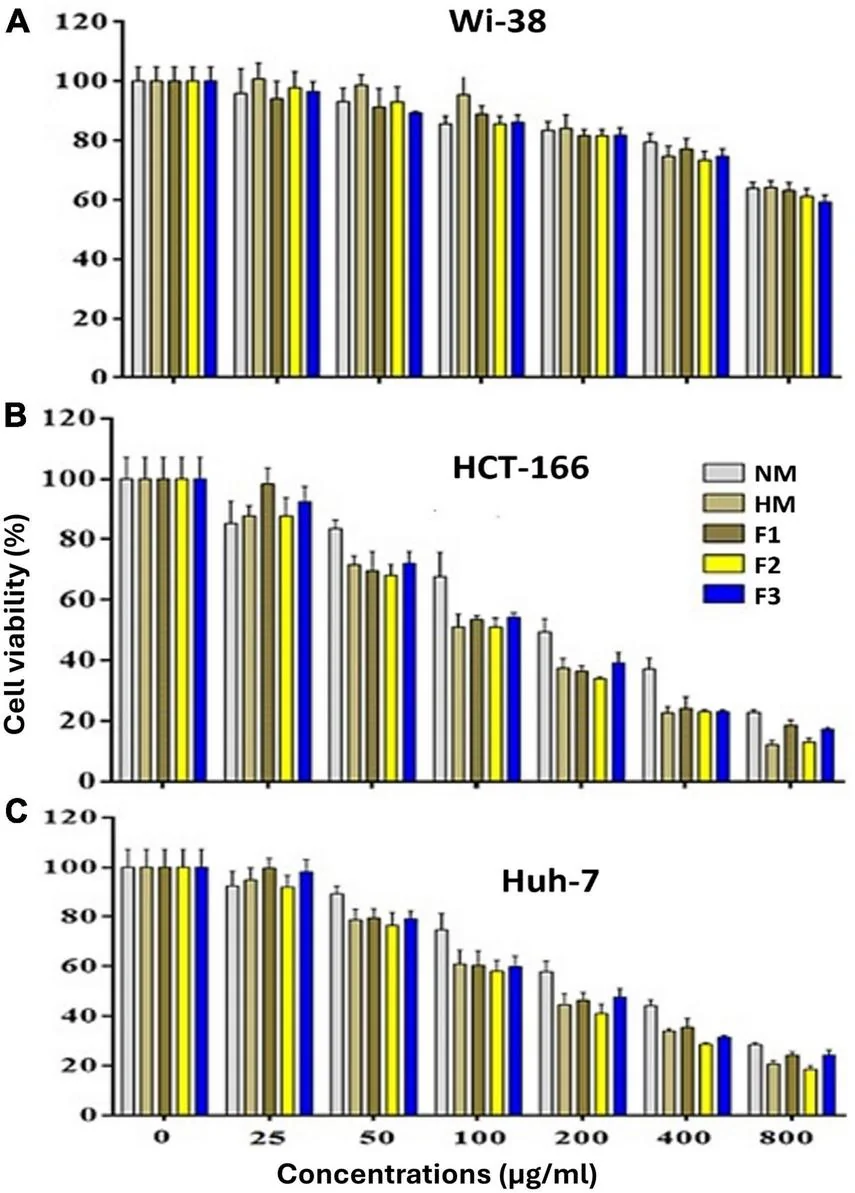
Effect of the native melittin (NM), hydrolysate (HM) and their alcalse hydrolysis fractions (F1, F2 and F3(on the cell viability of **(A)** the human normal (Wi-38) cells, **(B)** human cancer (HCT 166) and **C)** Huh-7 cell lines after treatment for 72 h compared to untreated cells (expressed as Mean ± SE).

The results of the MTT assay showed that native melittin (NM), melittin hydrolysate (HM), and its fractions (F1, F2, and F3) significantly inhibited the cell proliferation (decreased the number of viable cells) of both cancer cells (Huh-7 and HCT 116) in a concentration-dependent manner as compared to the untreated cells. In addition, the tested peptides were found free of cytotoxic effect on normal cells (Wi-38) in contrast to cancer cells as shown in Figure 4. Data presented in Table 3 illustrated that 72 h treatment of melittin and its peptide fractions inhibited HCT 166 cell growth recording IC_50_ values in the range from 101 ± 5.56 to 194.9 ± 7.636 μg/ml, against 129.5 ± 6.087 to 272.6 ± 6.72 μg/ml in the case of Huh-7 cells, respectively. However, these values of IC_50_ were highly increased against the normal cells (Wi-38) to a range from 1149 ± 4.05 to 1329 ± 5.52 μg/ml. The obtained results reveal that colon carcinoma (HCT 116) cells were more sensitive to the tested compounds than liver carcinoma (Huh-7) cells as compared to normal lung fibroblast (Wi-38) cells. Furthermore, melittin and its peptide fractions had selectivity for both cancer cells, i.e., Huh-7 and HCT 116 cells, then normal Wi-38 cells.

**TABLE 3:** IC_50_ and selectivity index (SI) values of melittin and its peptide fractions (F1, F2 and F3) against tested cell lines after 72 h.

Tested compounds	IC_50_ (μg/ml) ± SD			SI	
	Wi-38	HCT 166	Huh-7	HCT 166	Huh-7
NM	1180 ± 6.3	194.9 ± 7.6	272.6 ± 6.7	6.05	4.3
HM	1285 ± 6.4	114.7 ± 5.4	142.5 ± 6.4	11.20	9.0
F1	1150 ± 6.0	103.4 ± 6.3	133.9 ± 6.3	11.12	8.6
F2	1329 ± 5.5	101.0 ± 5.6	129.5 ± 6.1	13.15	10.3
F3	1149 ± 4.1	113.8 ± 5.5	135.1 ± 5.8	10.09	8.5

SI indicates to the ratio of the IC_50_ value of normal cells versus the IC_50_ value of cancer cells.

**FIGURE 5:**
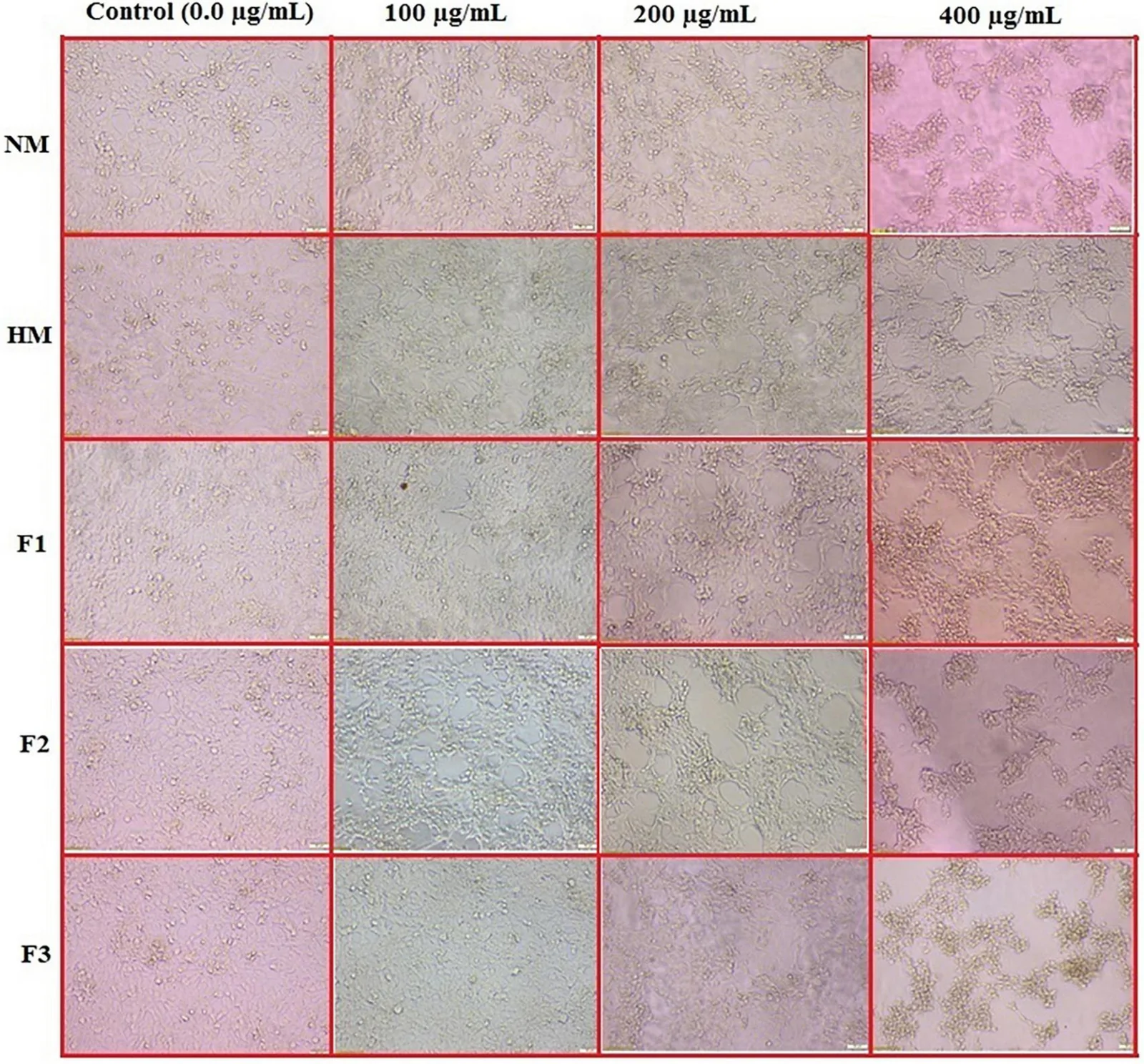
*In vitro* action of native melittin (NM), hydrolysate melittin (HM) and its peptide fractions (F1, F2 and F3) on the morphological alterations of HCT 166 cells as observed under a phase-contrast microscope (Scale bar = 500μm).

To detect the effect of NM, HM, and its fractions (F1, F2, and F3) on cell morphology, the HCT 166 cell line was used, and the data is recorded in Figure 5. The data confirmed that NM, HM and its fractions (F1, F2, and F3) inhibited HCT 116 cells growth in a dose and time-dependent manner with remarkable morphological changes in the treated cells, including, cell deformation, destruction of cell sheet, cytoplasmic condensation, and detaching from the surface of tissue culture and irregularity of cell shape, also some cells were gradually reduced in size and changed into a small round single cell shape when compared to untreated cells that were well adherent, homogeneously distributed in the culture field exhibiting a polygonal shape with distinct boundaries and homogenous cellular contents.

#### Cell migration inhibition assay

**FIGURE 6:**
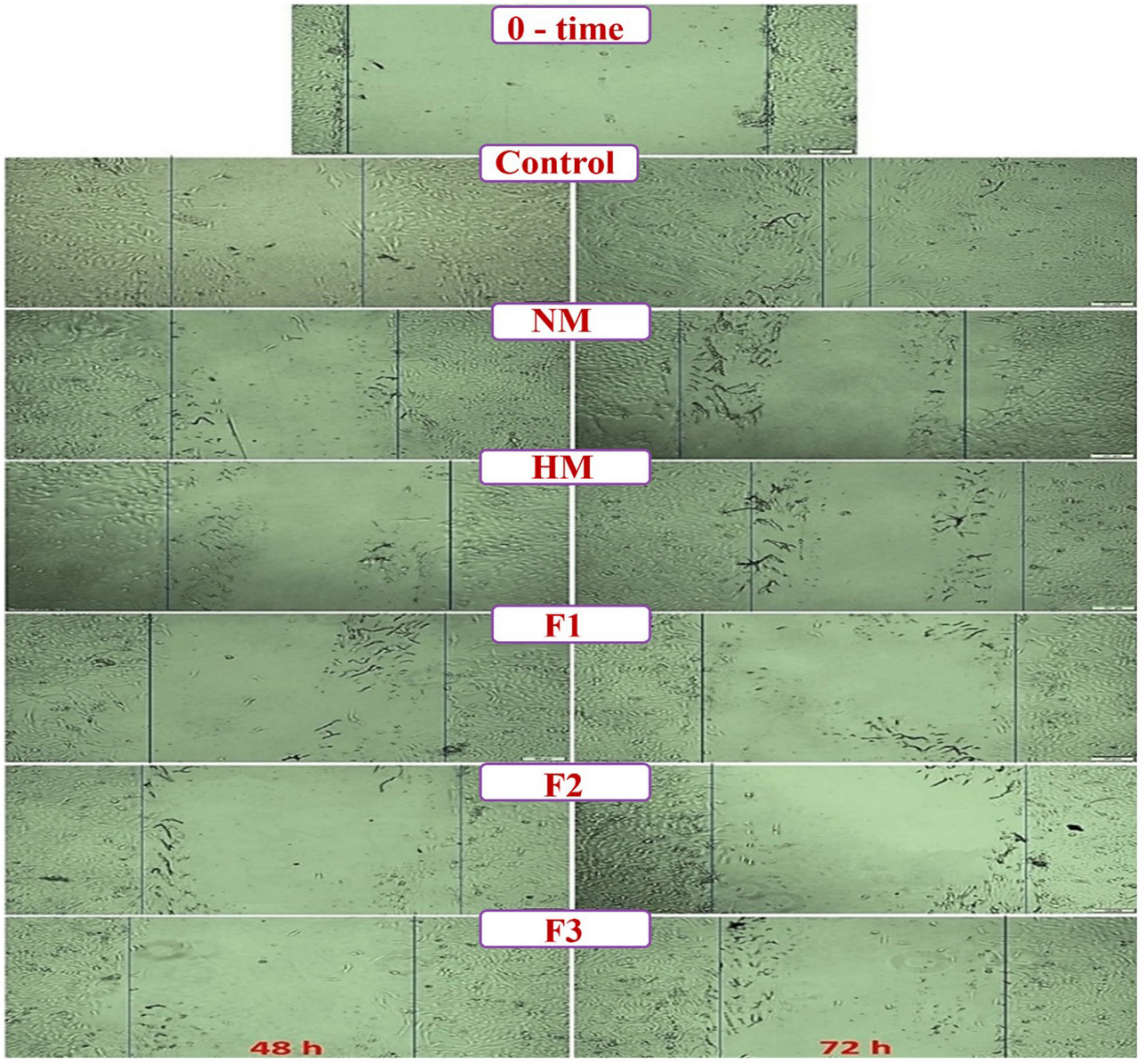
Microscopic micrographs of the scratched wound area in the treated Huh-7 cells with melittin, melittin hydrolysate (HM) and its peptide fractions (F1, F2, and F3) as compared to untreated (blank) cells at 0, 48 and 72 h (Scale bar = 500μm). All values are demonstrated as mean ± SE. Melittin and its fractions are statistically significant at *p* < 0.05*, *p* < 0.005**, *p* < 0.0005***.

The wound healing test is a well-established *in vitro* tool for investigating collective cell migration in two dimensions. The cells move into the gap after being exposed to the cell-free zone. Figure 6 depicts a series of typical photos from a wound healing experiment performed on a Huh-7 monolayer. The results demonstrated that melittin and its fractions had an anti-migration impact on Huh-7 cells after 48 and 72 hours, compared to the control cells. On the one hand, cells in the control treatment migrated to the cell-free region by more than 40% after 48 hours, whereas cell migration increased to almost 85% after 72 hours. On the other hand, melittin demonstrated anti-migration activity amounting to 76.25 ± 2.3 and 82.92 ± 1.63% after 48 and 72 h, respectively. The anti-migration impact of melittin fractions ranged from 81.42 ± 2.54% to 89.105 ± 1.53%. These data revealed that there were significant differences between the treatments when compared to the control. Moreover, the peptide F2 treatment showed the highest anti-migration effects impact as shown in Figure 6.

## Discussion

The current study involved the isolation of melittin from bee venom, followed by its hydrolysis using alcalase to produce novel bioactive peptides. These peptides were then assessed for their antibacterial and anticancer properties. One of four processes—solvent extraction, chemical processing, enzymatic hydrolysis, and microbial fermentation of dietary proteins—can be used to create bioactive peptides (Alasalvar et al., 2011). In the food and pharmaceutical industries, enzymatic hydrolysis is favored because other processes can leave harmful compounds or traces of organic solvents in the final products. Within the original protein sequences, the potentially bioactive peptides are inactive. So, liberating them by enzymatic hydrolysis may trigger their several physiological activities (Vercruysse et al., 2005). This fact applied to the current results, since hydrolyzed melittin (HM) and its fraction F1 frequently exhibited more effective actions across many bacterial strains than the native melittin (NM). The MIC of HM and its isolated fractions, especially F1 were considerably lower against most studied bacteria than NM. The inhibition zones induced by studied substances indicate also the issued fractions were more biologically active than the parent unhydrolyzed molecule (NM). This indicates that the hydrolysis process could liberate the active peptides from the parent molecules exhibiting more pronounced biological activities.

The biological activity of protein hydrolysates is influenced by many factors, e.g., the protein substrate, the proteolytic enzyme’s specificity, the hydrolysis conditions, and the degree of hydrolysis (Kristinsson and Rasco, 2000). According to the ESI-MS results, the majority of peptides released were basic and hydrophobic. The higher relative contents of hydrophobic peptide in the hydrolysate are mainly due to the amino acid composition of melittin, which is a cationic peptide with a hydrophobic nature and free of acidic amino acids and secondly because of the alcalase-specific activity, which produces mainly peptides with general hydrophobic characteristics (O’Connor et al., 2022). The great content of the hydrophobic peptides and the basic peptides in the melittin hydrolysate may affect the biological activity of this hydrolysate, as both hydrophobicity and net charge determine the antimicrobial activity of the peptide (Hancock et al., 2006). Basic or hydrophobic amino acids are used to obtain peptides with maximum activity (Jenssen et al., 2008). The content of hydrophobic amino acids in the active peptides was reported to be directly related to their antioxidant activity (Olagunju et al., 2018). It has been declared that hydrophobic amino acids, e.g., Leu, Ile, and Ala, contributed greatly to the peptide antioxidant activity (Mirzaei et al., 2020). Aromatic and hydrophobic amino acids significantly contribute to the radical scavenging of peptides by enhancing their interactions with lipids or acting as potent proton/ hydrogen donors (Sanjukta et al., 2021). Some AMPs exhibited anticancer activity with a mode of action compared with normal cells (Gaspar based on the alteration of membrane composition (Gabernet et al., 2016). The hydrophobic penetration peptide (HPP) conjugated stealth liposomes significantly enhanced the intracellular delivery of Doxorubicin (an anticancer drug) against heterogeneous breast cancer cells and increased the cytotoxic activity in vitro via a hydrophobic interaction (Cai et al., 2014). The importance of hydrophobicity and alkalinity in anticancer drugs has already been realized. Cationic lipids have been employed as gene delivery vectors (Ko et al., 2009), confirming the importance of these two traits supporting the transfection capacity of the drug (Xu et al., 2019). The cytotoxic action of cationic peptides on both microorganisms and neoplastic cells is largely believed to be a function of their amphipathic nature and secondary structure (Powers and Hancock, 2003; Schweizer, 2009).

The findings result of antimicrobial testing for melittin, HM, and its peptide fractions F1, F2, and F3 demonstrate broad-spectrum efficacy against tested bacteria. These results suggest that HM is the most active substance examined since it exhibited bigger inhibition zones and lower MIC against various bacterial strains. This finding corresponds well with Osman et al. (2016b), who found that enzymatic hydrolysis of goat whey, prepared from goat milk using alcalase enhanced its biological activity. Also, Abdel-Hamid et al. (2016) have shown that the Camel whey (CW), hydrolysed with papain from *Carica papaya*, exhibited significantly higher antibacterial activity than the unhydrolyzed CW. The antibacterial activity of melittin and its peptide fractions, as demonstrated in the study, presents a promising avenue for combating bacterial infections. The enhanced efficacy of HM over NM and its fractions could be attributed to one key factor, which is the modification of melittin structure, reducing the proteolytic degradation by bacterial enzymes. Askari et al. (2021) reported that the changes made to melittin, particularly through N-terminal amino acid conjugation, play a crucial role in enhancing its antibacterial activity. Recent research has examined the antimicrobial properties of melittin, an inherent antimicrobial peptide discovered in bee venom. The broad-spectrum antimicrobial activity of melittin makes it a highly prospective option in this field of antibiotic development. Due to its amphiphilic structure, melittin can facilitate efficient interactions with bacterial membranes, resulting in bactericidal effects. Melittin was demonstrated to exhibit significant antibacterial properties against a wide range of strains, including *Staphylococcus aureus*, even at low toxic concentrations (Huang et al., 2024). Another recent study has highlighted the multifaceted nature of melittin as a triple-action agent. This triple-action mechanism involves disrupting bacterial membranes, inhibiting biofilm formation, and modulating immune responses to enhance antimicrobial effects (Yang et al., 2024).

The present study assessed the effectiveness of melittin, HM, and F1, F2, and F3 on *S. aureus* and *P. aeruginosa* biofilms *in vitro*. Inhibitory activity against biofilm formation was highest for fraction F1 against both *S. aureus* and *P. aeruginosa*. Since microbial cells inhabit many environmental niches, biofilms are pervasive and can be observed in human tissues, organs, medical devices, and food processing equipment. Eliminating a bacterial infection becomes exceedingly challenging once it is established (Mishra et al., 2020). Their biofilms erect formidable obstacles that evade the host’s immune system and antibiotics, potentially leading to the development of antibiotic-resistant strains and concerning rates of illness and death. Hence, it is necessary to discover and develop novel antibacterial compounds to reinforce the clinical treatment of bacterial infections and prevent biofilm formation (Krukiewicz et al., 2022). These results suggest that fraction F1 possesses strong antibiofilm properties, declaring its effectiveness against biofilm formation by the two bacteria. Peptides derived from melittin demonstrated superior efficacy in impeding biofilm formation compared to single melittin, probably due to several critical factors. Peptides obtained from melittin are subjected to structural modifications that augment their effectiveness in impeding biofilm formation through improved interaction sites with bacterial membranes or other biofilm development-related targets. The peptides exhibit enhanced specificity towards biofilm formation-critical targets, including signaling molecules and enzymes, in contrast to the parent compound. This characteristic enables more targeting inhibition. Increased stability of peptides derived from melittin safeguards them against degradation by proteases and other bacterial environmental factors. Thus, their activity against biofilm-forming bacteria is maintained for an extended period. The combined influence of these elements results in the remarkable efficacy of peptides derived from melittin in impeding the formation of biofilms, rendering them (Mirzaei et al., 2022; Yang et al., 2024).

The cationic nature of melittin may qualify it to play a crucial role in the disruption of cancer cell membranes through electrostatic attraction between the negatively charged membrane components of cancer cells and the positive charges of the alkaline peptides (Adler-Nissen, 1979). Because of its excellent immunomodulatory effects, antitumor potency, and its ability to overcome tumor drug resistance, melittin, therefore, represents a promising agent for cancer treatment (Yu et al., 2023). Moreover, a previous study showed that melittin treatment induces apoptosis in human gastric cancer (SGC-7901) cells via modulating mitochondria pathways. These adjustments improved apoptotic influencing aspects, endonuclease G, and cytochrome C release, accompanied by caspase-3 activation and apoptosis in SGC-7901 cells (Kong et al., 2016). The present results demonstrated that peptides derived from melittin had superior efficacy against cancer Huh-7 and HCT 116 cells compared to the native melittin. The small size and concentrated positive charges on the melittin-liberated peptides may enhance their potency in preventing the replication of cancer cells through interaction with cell membranes (Halim et al., 2018). Furthermore, the hydrophobicity of peptides is the primary factor determining the peptide’s ability for cancer inhibition. Hydrophobic peptides can improve the interactions between anti-cancer peptides and the membrane bilayers on the outer leaflets of tumor cells (Khongdetch et al., 2022). Based on the results obtained on IC_50_ and selectivity index values of this study, it seems that F2 fraction is the most promising compound, being the most inhibitory against both cancer cells (Huh-7 & HCT 116), and the safest peptide fraction against normal cells. This vigorous anticancer activity of F2 may be due to a high percentage of hydrophobic amino acids with anticancer properties against cell proliferation for both cancer cells. A study by Siriwat et al. (2023) found that the protein hydrolysates prepared from *Wolffia globose* using Alcalase and Protamex showed an inhibitory effect on the growth of the cancer A2780 cell line with less than 50% at the maximum dose of the protein hydrolysates. Meanwhile, the protein hydrolysates showed no cytotoxicity to normal cells. The present results confirmed the anticancer potential of melittin and its peptide fractions through *in vitro* migration and invasion assays using Huh-7 cells. In particular, peptides derived from melittin showed more robust anticancer efficacy than native melittin.

## Conclusion

Melittin was isolated from bee venom (BV) of honeybee *Apis mellifera*, hydrolyzed by alcalase at pH 7 and 50°C for 60-min, giving out melittin hydrolysate (HM), which was fractionated by Sephadex G-25 gel filtration chromatography into three fractions (F1, F2, and F3). The results showed that native melittin (NM), melittin hydrolysate (HM), and its peptide fractions F1, F2, and F3 have a wide range of biological activities, including antibacterial, anti-biofilm, anticancer, and anti-migration. However, enzymatic hydrolysis of melittin enhanced all tested biological activities. From the antibacterial results, melittin hydrolysate (HM) and fraction F1 exhibited more effective antibacterial action than the native melittin (NM). Likewise, fraction F1 displayed the highest activity against biofilm formation by *S. aureus* and *P. aeruginosa* at 1.0 MIC concentration. Alternatively, the anticancer results demonstrated that peptides derived from melittin had superior efficacy against cancer Huh-7 and HCT 116 cells in comparison to native melittin. F2 fraction is the most effective peptide against both cancer Huh-7 & HCT 116 cells. Furthermore, all tested peptides could selectively inhibit cancer cells and did not affect the viability of normal lung fibroblast (Wi-38) cells. Similarly, fraction F2 showed the highest anti-migration impact against Huh-7 cells. These results confirmed the anticancer potential of melittin and its peptide fractions through *in vitro* migration. The present results are highly encouraging for the future utilization of melittin hydrolysates as novel bioactive peptides for promising antimicrobial and anticancer agents against human liver (Huh-7) and colon (HCT 166) cancer cells. Further studies are required to define the most active peptides and to achieve better biological actions and long-term safety.

## Data availability statement

The original contributions presented in the study are included in the article/Supplementary material, further inquiries can be directed to the corresponding authors.

## Author contributions

SE-D: Conceptualization, Data curation, Validation, Writing−original draft, Writing−review and editing. MK: Investigation, Methodology, Visualization, Writing−original draft, Writing−review and editing. MHS: Investigation, Methodology, Validation, Writing−original draft, Writing−review and editing. EE-F: Formal analysis, Investigation, Methodology, Writing−original draft, Writing−review and editing. AO: Conceptualization, Data curation, Methodology, Writing−original draft, Writing−review and editing. MS: Conceptualization, Data curation, Formal analysis, Supervision, Writing – original draft, Writing−review and editing. BS: Conceptualization, Data curation, Funding acquisition, Writing−original draft, Writing−review and editing.

## References

[B1] Abdel-Hamid M. Goda H. A. De Gobba C. Jenssen H. Osman A. J. (2016). Antibacterial activity of papain hydrolysed camel whey and its fractions. *Int. Dairy J.* 61 91–98. 10.1016/j.idairyj.2016.04.004

[B2] Abdel-Hamid M. Otte J. De Gobba C. Osman A. Hamad E. (2017). Angiotensin I-converting enzyme inhibitory activity and antioxidant capacity of bioactive peptides derived from enzymatic hydrolysis of buffalo milk proteins. *Int. Dairy J.* 66 91–98. 10.1016/j.idairyj.2016.11.006

[B3] AbdelMageed M. Ali H. Olsson L. Lindmark G. Hammarström M.-L. Hammarström S. (2019). The chemokine CXCL16 is a new biomarker for lymph node analysis of colon cancer outcome. *Int. J. Mol. Sci.* 20:5793. 10.3390/ijms20225793 31752131 PMC6888697

[B4] AbdelMageed M. Ismail H.T.H. Olsson L. Lindmark G. Hammarström M.-L. Hammarström S. (2021). Clinical significance of stem cell biomarkers epcam, lgr5 and lgr4 mrna levels in lymph nodes of colon cancer patients. *Int. J. Mol. Sci.* 23:403. 10.3390/ijms23010403 35008827 PMC8745090

[B5] Abdel-Shafi S. Al-Mohammadi A. R. Osman A. Enan G. Abdel-Hameid S. Sitohy M. (2019a). Characterization and antibacterial activity of 7S and 11S globulins isolated from cowpea seed protein. *Molecules* 24:1082. 10.3390/molecules24061082 30893826 PMC6471422

[B6] Abdel-Shafi S. Osman A. Al-Mohammadi A. R. Enan G. Kamal N. Sitohy M. (2019b). Biochemical, biological characteristics and antibacterial activity of glycoprotein extracted from the epidermal mucus of African catfish (Clarias gariepinus). *Int. J. Biol. Macromol.* 138 773–780. 10.1016/j.ijbiomac.2019.07.150 31351952

[B7] Abdel-Shafi S. Osman A. Enan G. El-Nemer M. Sitohy M. (2016). Antibacterial activity of methylated egg white proteins against pathogenic G+ and G- bacteria matching antibiotics. *SpringerPlus*. 5 983–995. 10.1186/s40064-016-2625-3 27429892 PMC4932028

[B8] Abu-Serie M.M. El-Fakharany E.M. (2017). Efficiency of novel nanocombinations of bovine milk proteins (lactoperoxidase and lactoferrin) for combating different human cancer cell lines. *Sci. Rep.* 7:16769. 10.1038/s41598-017-16962-6 29196676 PMC5711920

[B9] Adler-Nissen J. (1979). Determination of the degree of hydrolysis of food protein hydrolysates by trinitrobenzenesulfonic acid. *J. Agric. Food Chem.* 27 1256–1262. 10.1021/jf60226a042 544653

[B10] Alasalvar C. Miyashita K. Shahidi F. Wanasundara U. (2011). *Handbook of Seafood Quality, Safety and Health Applications*. Hoboken, NJ: John Wiley & Sons.

[B11] Ali H. AbdelMageed M. Olsson L. Israelsson A. Lindmark G. Hammarström M. L. (2019). Utility of G protein-coupled receptor 35 expression for predicting outcome in colon cancer. *Tumor Biol.* 41 1–11. 10.1177/1010428319858885 31250711

[B12] Ali H. Olsson L. Lindmark G. Hammarström M.-L. Hammarström S. Sitohy B. (2021). The myeloid cell biomarker EMR1 is ectopically expressed in colon cancer. *Tumor Biol.* 43 209–223. 10.3233/TUB-200082 34486997

[B13] Al-Mohammadi A.-R. Osman A. Enan G. Abdel-Shafi S. El-Nemer M. Sitohy M. (2020). Powerful antibacterial peptides from egg albumin hydrolysates. *Antibiotics* 9:901. 10.3390/antibiotics9120901 33322196 PMC7763489

[B14] Askari P. Namaei M.H. Ghazvini K. Hosseini M.J. (2021). In vitro and *in vivo* toxicity and antibacterial efficacy of melittin against clinical extensively drug-resistant bacteria. *BMC Pharmacol. Toxicol.* 22:42. 10.1186/s40360-021-00503-z 34261542 PMC8281584

[B15] Azevedo M.M. Pina-Vaz C. Baltazar F. (2020). Microbes and cancer: friends or faux? *Int. J. Mol. Sci.* 21:3115. 10.3390/ijms21093115 32354115 PMC7247677

[B16] Cai D. Gao W. He B. Dai W. Zhang H. Wang X. (2014). Hydrophobic penetrating peptide PFVYLI-modified stealth liposomes for doxorubicin delivery in breast cancer therapy. *Biomaterials* 35 2283–2294. 10.1016/j.biomaterials.2013.11.088 24360410

[B17] Dawoud N. T. El-Fakharany E. M. Abdallah A. E. El-Gendi H. Lotfy D. (2022). Synthesis, and docking studies of novel heterocycles incorporating the indazolylthiazole moiety as antimicrobial and anticancer agents. *Sci. Rep.* 12:3424. 10.1038/s41598-022-07456-1 35236889 PMC8891364

[B18] El-Didamony S. E. Amer R. I. El-Osaily G. H. (2022a). Formulation, characterization and cellular toxicity assessment of a novel bee-venom microsphere in prostate cancer treatment. *Sci. Rep.* 12:13213. 10.1038/s41598-022-17391-w 35918370 PMC9346107

[B19] El-Didamony S.E. Kalaba M.H. El-Fakharany E.M. Sultan M.H. Sharaf M.H. (2022b). Antifungal and antibiofilm activities of bee venom loaded on chitosan nanoparticles: a novel approach for combating fungal human pathogens. *World J. Microbiol. Biotechnol.* 38:244. 10.1007/s11274-022-03425-y 36280608 PMC9592658

[B20] El-Salhy M. Sitohy B. (2002). Triple therapy with octreotide, galanin and serotonin induces necrosis and increases apoptosis of a rat colon carcinoma. *Regul. Pept.* 108 55–62. 10.1016/S0167-0115(02)00106-4 12220727

[B21] El-Salhy M. Sitohy B. Norrgård Ö. (2003). Triple therapy with octreotide, galanin, and serotonin reduces the size and blood vessel density and increases apoptosis of a rat colon carcinoma. *Regul. Pept.* 111 145–152. 10.1016/S0167-0115(02)00280-X 12609762

[B22] El-Sherbiny G.M. Kalaba M.H. Sharaf M.H. Moghannem S.A. Radwan A.A. Askar A.A. (2022). Biogenic synthesis of CuO-NPs as nanotherapeutics approaches to overcome multidrug-resistant Staphylococcus aureus (MDRSA). *Artif. Cells Nanomed. Biotechnol.* 50 260–274. 10.1080/21691401.2022.2126492 36191138

[B23] Eltorky H. AbdelMageed M. Ismail H. Zahran F. Guirgis A. Olsson L. (2024). LGR6 is a prognostic biomarker for less differentiated tumors in lymph nodes of colon cancer patients. *Front. Oncol.* 14: 1393075. 10.3389/fonc.2024.1393075 38715790 PMC11074358

[B24] El-Zahar K. Chobert J.M. Sitohy M. Dalgalarrondo M. Haertlé T. (2003). Proteolytic degradation of ewe milk proteins during fermentation of yoghurts and storage. *Nahrung* 47 199–206. 10.1002/food.200390046 12866624

[B25] El-Zahar K. Sitohy M. Choiset Y. Metro F. Haertle T. Chobert J. (2004). Antimicrobial activity of ovine whey protein and their peptic hydrolysates. *Milchwissenschaft* 59 653–656.

[B26] Evans R.W. Williams J. (1980). The electrophoresis of transferrins in urea/polyacrylamide gels. *Biochem. J.* 189 541–546. 10.1042/bj1890541 7213345 PMC1162034

[B27] Fakhim-Zadeh K. (1998). Improved device for venom extraction. *Bee World* 79 52–56. 10.1080/0005772X.1998.11099379

[B28] Ferlay J. Colombet M. Soerjomataram I. Parkin D.M. Piñeros M. Znaor A. (2021). Cancer statistics for the year 2020: an overview. *Int. J. Cancer* 149 778–789. 10.1002/ijc.33588 33818764

[B29] Gabernet G. Müller A. Hiss J. Schneider G. (2016). Membranolytic anticancer peptides. *Medchemcomm* 7 2232–2245. 10.1039/C6MD00376A

[B30] Geitani R. Ayoub M.C. Touqui L. Karam S.D. (2019). Cationic antimicrobial peptides: alternatives and/or adjuvants to antibiotics active against methicillin-resistant Staphylococcus aureus and multidrug-resistant *Pseudomonas aeruginosa*. *BMC Microbiol.* 19:54. 10.1186/s12866-019-1416-8 30849936 PMC6408789

[B31] Guha S. Ferrie R. P. Ghimire J. Ventura C. R. Wu E. Sun L. (2021). Applications and evolution of melittin, the quintessential membrane active peptide. *Biochem. Pharmacol*. 193:114769. 10.1016/j.bcp.2021.114769 34543656 PMC9235364

[B32] Gupta R.K. Stangaciu S. (2014). “Apitherapy: holistic healing through the honeybee and bee products in countries with poor healthcare system,” in *Beekeeping for Poverty Alleviation and Livelihood Security*, eds Gupta R. K. Reybroeck W. van Veen J. W. Gupta A.. Springer: Dordrecht

[B33] Halim N.R.A. Azlan A. Yusof H.M. Sarbon N.M. (2018). Antioxidant and anticancer activities of enzymatic eel (monopterus sp) protein hydrolysate as influenced by different molecular weight. *Biocatal. Agric. Biotechnol.* 16 10–16. 10.1016/j.bcab.2018.06.006

[B34] Han H. Chen W. Yang J. Liang X. Wang Y. Li Q. (2018). Inhibition of cell proliferation and migration through nucleobase-modified polyamidoamine-mediated p53 delivery. *Int. J. Nanomedicine* 13 1297–1311. 10.2147/IJN.S146917 29563788 PMC5846749

[B35] Hancock R.E. Brown K.L. Mookherjee N. (2006). Host defence peptides from invertebrates–emerging antimicrobial strategies. *Immunobiology* 211 315–322. 10.1016/j.imbio.2005.10.017 16697922

[B36] Hassan S.A. Alazragi R.S. Salem N. (2021). Potential therapeutic effect of Bee venom on cisplatin-induced hepatotoxicity. *J. Pharm. Res. Int.* 33 200–210. 10.9734/jpri/2021/v33i29A31579 35024509

[B37] Huang S. Su G. Jiang S. Chen L. Huang J. Yang F. (2024). New N-terminal fatty-acid-modified melittin analogs with potent biological activity. *Int. J. Mol. Sci.* 25:867. 10.3390/ijms25020867 38255940 PMC10815238

[B38] Jenssen H. Fjell C.D. Cherkasov A. Hancock R. (2008). QSAR modeling and computer-aided design of antimicrobial peptides. *J. Pept. Sci.* 14 110–114. 10.1002/psc.908 17847019

[B39] Kalaba M.H. Sultan M.H. Elbahnasawy M.A. El-Didamony S.E. El Bakary N.M. Sharaf M. (2022). First report on isolation of Mucor bainieri from honeybees, Apis mellifera: characterization and biological activities. *Biotechnol. Rep.* 36:e00770. 10.1016/j.btre.2022.e00770 36338578 PMC9634281

[B40] Khabbaz R.F. Moseley R.R. Steiner R.J. Levitt A.M. Bell B.P. (2014). Challenges of infectious diseases in the USA. *Lancet* 384 53–63. 10.1016/S0140-6736(14)60890-4 24996590 PMC7137922

[B41] Khalifa R.A. Nasser M.S. Gomaa A.A. Osman N.M. Salem H.M. (2013). Resazurin microtiter assay plate method for detection of susceptibility of multidrug resistant Mycobacterium tuberculosis to second-line anti-tuberculous drugs. *J. Chest. Dis. Tuberc.* 62 241–247. 10.1016/j.ejcdt.2013.05.008

[B42] Khongdetch J. Laohakunjit N. Kaprasob R. (2022). King Boletus mushroom-derived bioactive protein hydrolysate: characterisation, antioxidant, ACE inhibitory and cytotoxic activities. *Int. J. Food Sci. Technol.* 57 1399–1410. 10.1111/ijfs.15100

[B43] Ko Y.T. Falcao C. Torchilin V.P. (2009). Cationic liposomes loaded with proapoptotic Peptide d-(KLAKLAK)2 and Bcl-2 antisense oligodeoxynucleotide G3139 for enhanced anticancer therapy. *Mol. Pharmaceutics* 6 971–977. 10.1021/mp900006h 19317442 PMC2705994

[B44] Kong G. M. Tao W. H. Diao Y. L. Fang P. H. Wang J. J. Bo P. Qian F. (2016). Melittin induces human gastric cancer cell apoptosis via activation of mitochondrial pathway. *World J. Gastroenterol.* 22 3186–3195. 10.3748/wjg.v22.i11.3186 27003995 PMC4789993

[B45] Kreil G. (1973). Structure of melittin isolated from two species of honey bees. *FEBS Lett.* 33 241–244. 10.1016/0014-5793(73)80202-9

[B46] Kristinsson H.G. Rasco B. (2000). Fish protein hydrolysates: production, biochemical, and functional properties. *Crit. Rev. Food Sci. Nutr.* 40 43–81. 10.1080/10408690091189266 10674201

[B47] Krukiewicz K. Kazek-Kęsik A. Brzychczy-Włoch M. Łos M.J. Ateba C.N. Mehrbod P. (2022). Recent advances in the control of clinically important biofilms. *Int. J. Mol. Sci.* 23:9526. 10.3390/ijms23179526 36076921 PMC9455909

[B48] Laemmli U.K. (1970). Cleavage of structural proteins during the assembly of the head of bacteriophage T4. *Nature* 227 680–685. 10.1038/227680a0 5432063

[B49] Mahgoub S. Osman A. Sitohy M. (2011). Inhibition of growth of pathogenic bacteria in raw milk by legume protein esters. *J. Food Prot.* 74 1475–1481. 10.4315/0362-028X.JFP-11-065 21902916

[B50] Mansoori B. Mohammadi A. Davudian S. Shirjang S. Baradaran B. (2017). The different mechanisms of cancer drug resistance: a brief review. *Adv. Pharm. Bull.* 7 339–348. 10.15171/apb.2017.041 29071215 PMC5651054

[B51] Mirzaei M. Mirdamadi S. Safavi M. Soleymanzadeh N. (2020). The stability of antioxidant and ACE-inhibitory peptides as influenced by peptide sequences. *LWT-Food Sci. Technol.* 130:109710. 10.1016/j.lwt.2020.109710

[B52] Mirzaei R. Alikhani M.Y. Arciola C.R. Sedighi I. Yousefimashouf R. Bagheri K.P. (2022). Prevention, inhibition, and degradation effects of melittin alone and in combination with vancomycin and rifampin against strong biofilm producer strains of methicillin-resistant Staphylococcus epidermidis. *Biomed. Pharmacother.* 147:112670. 10.1016/j.biopha.2022.112670 35123230

[B53] Mishra R. Panda A.K. De Mandal S. Shakeel M. Bisht S.S. Khan J. (2020). Natural anti-biofilm agents: strategies to control biofilm-forming pathogens. *Front. Microbiol.* 11:566325. 10.3389/fmicb.2020.566325 33193155 PMC7658412

[B54] Morens D.M. Fauci A.S. (2012). Emerging infectious diseases in 2012: 20 years after the institute of medicine report. *MBio* 3 e412–e494. 10.1128/mBio.00494-12 23232716 PMC3520107

[B55] Mosmann T. (1983). Rapid colorimetric assay for cellular growth and survival: application to proliferation and cytotoxicity assays. *J. Immunol. Methods* 65 55–63. 10.1016/0022-1759(83)90303-4 6606682

[B56] O’Connor J. Garcia-Vaquero M. Meaney S. Tiwari B. (2022). Bioactive peptides from algae: traditional and novel generation strategies, structure-function relationships, and bioinformatics as predictive tools for bioactivity. *Mar. Drugs* 20:317. 10.3390/md20050317 35621968 PMC9145204

[B57] Olagunju A.I. Omoba O.S. Enujiugha V.N. Alashi A.M. Aluko R.E. (2018). Pigeon pea enzymatic protein hydrolysates and ultrafiltration peptide fractions as potential sources of antioxidant peptides: an *in vitro* study. *LWT-Food Sci. Technol.* 97 269–278. 10.1016/j.lwt.2018.07.003

[B58] Olsson L. Lindmark G. Hammarström M. L. Hammarström S. Sitohy B. (2020). Evaluating macrophage migration inhibitory factor 1 expression as a prognostic biomarker in colon cancer. *Tumor Biol.* 42:e23277. 10.1177/1010428320924524 32515296

[B59] Osman A. El-Didamony G. Sitohy M. Khalifa M. Enan G. (2016a). Soybean glycinin basic subunit inhibits methicillin resistant-vancomycin intermediate Staphylococcus aureus (MRSA-VISA) *in vitro*. *Int. J. Appl. Res. Nat. Prod.* 9 17–26.

[B60] Osman A. Enan G. Al-Mohammadi A.-R. Abdel-Shafi S. Abdel-Hameid S. Sitohy M.Z. (2021a). Antibacterial peptides produced by Alcalase from cowpea seed proteins. *Antibiotics* 10:870. 10.3390/antibiotics10070870 34356791 PMC8300757

[B61] Osman A. Goda H.A. Abdel-Hamid M. Badran S.M. Otte J. (2016b). Antibacterial peptides generated by Alcalase hydrolysis of goat whey. *LWT-Food Sci. Technol.* 65 480–486. 10.1016/j.lwt.2015.08.043

[B62] Osman A. Imbabi T.A. El-Hadary A. Sabeq I.I. Edris S.N. Merwad A.-R. (2021b). Health aspects, growth performance, and meat quality of rabbits receiving diets supplemented with lettuce fertilized with whey protein hydrolysate substituting nitrate. *Biomolecules* 11:835. 10.3390/biom11060835 34205142 PMC8227087

[B63] Pérez-Delgado O. Espinoza-Culupú A.O. López-López E. (2023). Antimicrobial activity of Apis mellifera Bee Venom collected in Northern Peru. *Antibiotics* 12:779. 10.3390/antibiotics12040779 37107142 PMC10135115

[B64] Powers J.-P.S. Hancock R.E.W. (2003). The relationship between peptide structure and antibacterial activity. *Peptides* 24 1681–1691. 10.1016/j.peptides.2003.08.023 15019199

[B65] Rady I. Siddiqui I. A. Rady M. Mukhtar H. (2017). Melittin, a major peptide component of bee venom, and its conjugates in cancer therapy. *Cancer Lett.* 402 16–31. 10.1016/j.canlet.2017.05.010 28536009 PMC5682937

[B66] Rashad Y. Olsson L. Israelsson A. Öberg Å Lindmark G. Hammarström M.-L. (2018). Lymph node CXCL17 messenger RNA: a new prognostic biomarker for colon cancer. *Tumor Biol.* 40:1010428318799251. 10.1177/1010428318799251 30198422

[B67] Rayaprolu S. J. Hettiarachchy N. S. Horax R. Phillips G. K. Mahendran M. Chen P. (2017). Soybean peptide fractions inhibit human blood, breast and prostate cancer cell proliferation. *J. Food Sci. Technol.* 54 38–44. 10.1007/s13197-016-2426-2 28242901 PMC5305699

[B68] Rayaprolu S. Hettiarachchy N. Horax R. Satchithanandam E. Chen P. Mauromoustakos A. (2015). Amino acid profiles of 44 soybean lines and ACE-I inhibitory activities of peptide fractions from selected lines. *J. Am. Oil Chem. Soc.* 92 1023–1033. 10.1007/s11746-015-2655-y

[B69] Renzo B. Tappin M. Annalisa P. Harvey T. Carver J. Campbell I. (1988). The structure of melittin. *Eur. J. Biochem.* 173 139–146. 10.1111/j.1432-1033.1988.tb13977.x 3356186

[B70] Ritchie H. Spooner F. Roser M. (2018). *Causes of Death. Our World in Data.* Available online at: https://ourworldindata.org/causes-of-death

[B71] Sanjukta S. Padhi S. Sarkar P. Singh S. P. Sahoo D. Rai A. (2021). Production, characterization and molecular docking of antioxidant peptides from peptidome of kinema fermented with proteolytic Bacillus spp. *Food Res. Int.* 141:110161. 10.1016/j.foodres.2021.110161 33642021

[B72] Schweizer F. (2009). Cationic amphiphilic peptides with cancer-selective toxicity. *Eur. J. Pharmacol.* 625 190–194. 10.1016/j.ejphar.2009.08.043 19835863

[B73] Siriwat W. Ungwiwatkul S. Unban K. Laokuldilok T. Klunklin W. Tangjaidee P. (2023). Extraction, Enzymatic Modification, and Anti-Cancer Potential of an Alternative Plant-Based Protein from Wolffia globosa. *Foods* 12:3815. 10.3390/foods12203815 37893708 PMC10606862

[B74] Sitohy B. El-Salhy M. (2001). Colonic endocrine cells in rats with chemically induced colon carcinoma. *Histol. Histopathol.* 16 833–838. 10.14670/HH-16.833 11510974

[B75] Sitohy B. El-Salhy M. (2002). Changes in the colonic enteric nervous system in rats with chemically induced colon dysplasia and carcinoma. *Acta Oncol.* 41 543–549. 10.1080/028418602320807810 12546527

[B76] Sitohy B. El-Salhy M. (2003). A comparison between double and triple therapies of octreotide, galanin and serotonin on a rat colon carcinoma. *Histol. Histopathol.* 18 103–110. 10.14670/HH-18.103 12507289

[B77] Sitohy B. Chang S. Sciuto T. E. Masse E. Shen M. Kang P. M. (2017). Early actions of anti–vascular endothelial growth factor/vascular endothelial growth factor receptor drugs on angiogenic blood vessels. *Am. J. Pathol.* 187 2337–2347. 10.1016/j.ajpath.2017.06.010 28736316 PMC5809587

[B78] Sitohy M. Z. Mahgoub S. A. Osman A. O. (2012). *In vitro* and *in situ* antimicrobial action and mechanism of glycinin and its basic subunit. *Int. J. Food Microbiol.* 154 19–29. 10.1016/j.ijfoodmicro.2011.12.004 22236762

[B79] Sitohy M. Chobert J. M. Gaudin J. C. Renac T. Haertlé T. (2002). When positively charged milk proteins can bind to DNA. *J. Food Biochem.* 26 511–532. 10.1111/j.1745-4514.2002.tb00770.x

[B80] Sitohy M. Chobert J.-M. Haertlé T. (2001b). Study of the formation of complexes between DNA and esterified dairy proteins. *Int. Dairy J.* 11 873–883. 10.1016/S0958-6946(01)00124-8

[B81] Sitohy M. Chobert J.-M. Gaudin J.-C. Haertlé T. (2001a). Esterified milk proteins inhibit DNA replication *in vitro*. *Int. J. Biol. Macromol.* 29 259–266. 10.1016/S0141-8130(01)00176-3 11718822

[B82] Sitohy M. Chobert J.-M. Karwowska U. Gozdzicka-Jozefiak A. Haertlé T. (2006). Inhibition of bacteriophage M13 replication with esterified milk proteins. *J. Agric. Food Chem.* 54 3800–3806. 10.1021/jf0531757 16719499

[B83] Son D. J. Lee J. W. Lee Y. H. Song H. S. Lee C. K. Hong J. T. (2007). Therapeutic application of anti-arthritis, pain-releasing, and anti-cancer effects of bee venom and its constituent compounds. *Pharmacol. Therapeut.* 115 246–270. 10.1016/j.pharmthera.2007.04.004 17555825

[B84] Taher F. Moselhy W. Mohamed A. Didamony S. Metwalley K. Zayed A. (2017). Preparation and characterization of shrimp derived chitosan and evaluation of its efficiency as bee venom delivery for cancer treatment. *Int. J. Adv. Res.* 5 370–388. 10.21474/IJAR01/4122

[B85] Uversky V. N. El-Fakharany E. M. Abu-Serie M. M. Almehdar H. A. Redwan E. M. (2017). Divergent anticancer activity of free and formulated camel milk α-lactalbumin. *Cancer Investig.* 35 610–623. 10.1080/07357907.2017.1373783 28949782

[B86] van Duin D. Paterson D. (2020). Multidrug-resistant bacteria in the community: an update. *Infect. Dis. Clin.* 34 709–722. 10.1016/j.idc.2020.08.002 33011046 PMC8713071

[B87] Vercruysse L. Van Camp J. Smagghe G. (2005). ACE inhibitory peptides derived from enzymatic hydrolysates of animal muscle protein: a review. *J. Agri. . Food Chem.* 53 8106–8115. 10.1021/jf0508908 16218651

[B88] Vogel H. Jähnig F. (1986). The structure of melittin in membranes. *Biophys. J.* 50 573–582. 10.1016/S0006-3495(86)83497-X 3779000 PMC1329835

[B89] Walaa A. M. Samia E. Fatma A. T. Aly F. M. Karima M. J. (2017). Evaluation of anticancer potentials of bee free venom and chitosan nano-conjugated one: *In vitro* study. *Int. J. Sci. Res. Mang.* 5 5253–5262.

[B90] Waldman E. A. Sato A. P. (2016). Path of infectious diseases in Brazil in the last 50 years: an ongoing challenge. *Rev. Saude Publica.* 50:68. 10.1590/s1518-8787.2016050000232 28099652 PMC5152805

[B91] Wayne P. (2015). *Performance Standards for Antimicrobial Susceptibility Testing.* Pennsylvania: Clinical and Laboratory Standards Institute

[B92] Wilson M. K. Karakasis K. Oza A. (2015). Outcomes and endpoints in trials of cancer treatment: the past, present, and future. *Lancet. Oncol.* 16 e32–e42. 10.1016/S1470-2045(14)70375-4 25638553

[B93] Xu X. Liu A. Bai Y. Li Y. Zhang C. Cui S. (2019). Co-delivery of resveratrol and p53 gene via peptide cationic liposomal nanocarrier for the synergistic treatment of cervical cancer and breast cancer cells. *J. Drug Deliv. Sci. Technol.* 51 746–753. 10.1016/j.jddst.2018.05.008

[B94] Yang H. Ma R. Chen J. Xie Q. Luo W. Sun P. (2024). Discovery of melittin as triple-action agent: broad-spectrum antibacterial, anti-biofilm, and potential anti-quorum sensing activities. *Molecules* 29:558. 10.3390/molecules29030558 38338303 PMC10856726

[B95] Yu X. Jia S. Yu S. Chen Y. Zhang C. Chen H. (2023). Recent advances in melittin-based nanoparticles for antitumor treatment: from mechanisms to targeted delivery strategies. *J. Nanobiotechnology.* 21:454. 10.1186/s12951-023-02223-4 38017537 PMC10685715

[B96] Zhang H.-Q. Sun C. (2024). The current landscape of the antimicrobial peptide melittin and its therapeutic potential. *Front. Immunol.* 15:1326033. 10.3389/fimmu.2024.1326033 38318188 PMC10838977

