# Correction: Melittin alcalase-hydrolysate: a novel chemically characterized multifunctional bioagent; antibacterial, anti-biofilm and anticancer

**DOI:** 10.3389/fmicb.2026.1988804

**Published:** 2026-09-24

**Authors:** Samia E. El-Didamony, Mohamed H. Kalaba, Mohamed H. Sharaf, Esmail M. El-Fakharany, Ali Osman, Mahmoud Sitohy, Basel Sitohy

**Affiliations:** 1 Department of Zoology and Entomology, Faculty of Science, Al-Azhar University (Girls), Nasr City, Egypt; 2 Department of Botany and Microbiology, Faculty of Science, Al-Azhar University (Boys), Cairo, Egypt; 3 Protein Research Department, Genetic Engineering and Biotechnology Research Institute (GEBRI), City of Scientific Research and Technological Applications (SRTA-City), New Borg Al-Arab City, Alexandria, Egypt; 4 Pharmaceutical and Fermentation Industries Development Center, City of Scientific Research and Technological Applications (SRTA-City), New Borg Al-Arab City, Alexandria, Egypt; 5 Pharos University in Alexandria, Alexandria, Egypt; 6 Department of Biochemistry, Faculty of Agriculture, Zagazig University, Zagazig, Egypt; 7 Department of Clinical Microbiology, Infection, and Immunology, Umeå University, Umeå, Sweden; 8 Department of Diagnostics and Intervention, Oncology, Umeå University, Umeå, Sweden

**Keywords:** bee venom, melittin, *Apis mellifera*, antibacterial activity, anticancer activity, enzymatic hydrolysis, electro-spray ionization, wound healing assay

In **Materials and methods**, *Electro-Spray-Ionization-Mass-Spectrometry (ESI-MS)*, page 3, the manuscript mistakenly referred to “BLG” (beta-lactoglobulin). The correct protein is melittin.

The incorrect sentence read: “The masses of the resulting peptides were matched to the original amino acid sequence of BLG registered in https://www.uniprot.org/.”

The corrected sentence should read: “The masses of the resulting peptides were matched to the original amino acid sequence of melittin registered in https://www.uniprot.org/.”

In **Conclusion**, page 10, the manuscript incorrectly stated that the “2-h hydrolysate” was fractionated into F1–F3.

The incorrect sentence read: “Melittin was isolated from bee venom (BV) of honeybee Apis mellifera, hydrolyzed by alcalase at pH 7 and 50 °C for two hours, giving out melittin hydrolysate (HM), which was fractionated by Sephadex G-25 gel filtration chromatography into three fractions (F1, F2, and F3).”

The correct sentence should read: “Melittin was isolated from bee venom (BV) of honeybee *Apis mellifera*, hydrolyzed by alcalase at pH 7 and 50 °C for 60-min, giving out melittin hydrolysate (HM), which was fractionated by Sephadex G-25 gel filtration chromatography into three fractions (F1, F2, and F3).”

The original version of this article has been updated.

