# Supplementary material for: Melittin alcalase-hydrolysate: a novel chemically characterized multifunctional bioagent; antibacterial, anti-biofilm and anticancer
Source: Front Microbiol. 2024 Jul 17;15:1419917. doi: 10.3389/fmicb.2024.1419917 (PMC11293514; doi:10.3389/fmicb.2024.1419917)
Supplement: Supplementary file 1 [file Data_Sheet_1.docx]

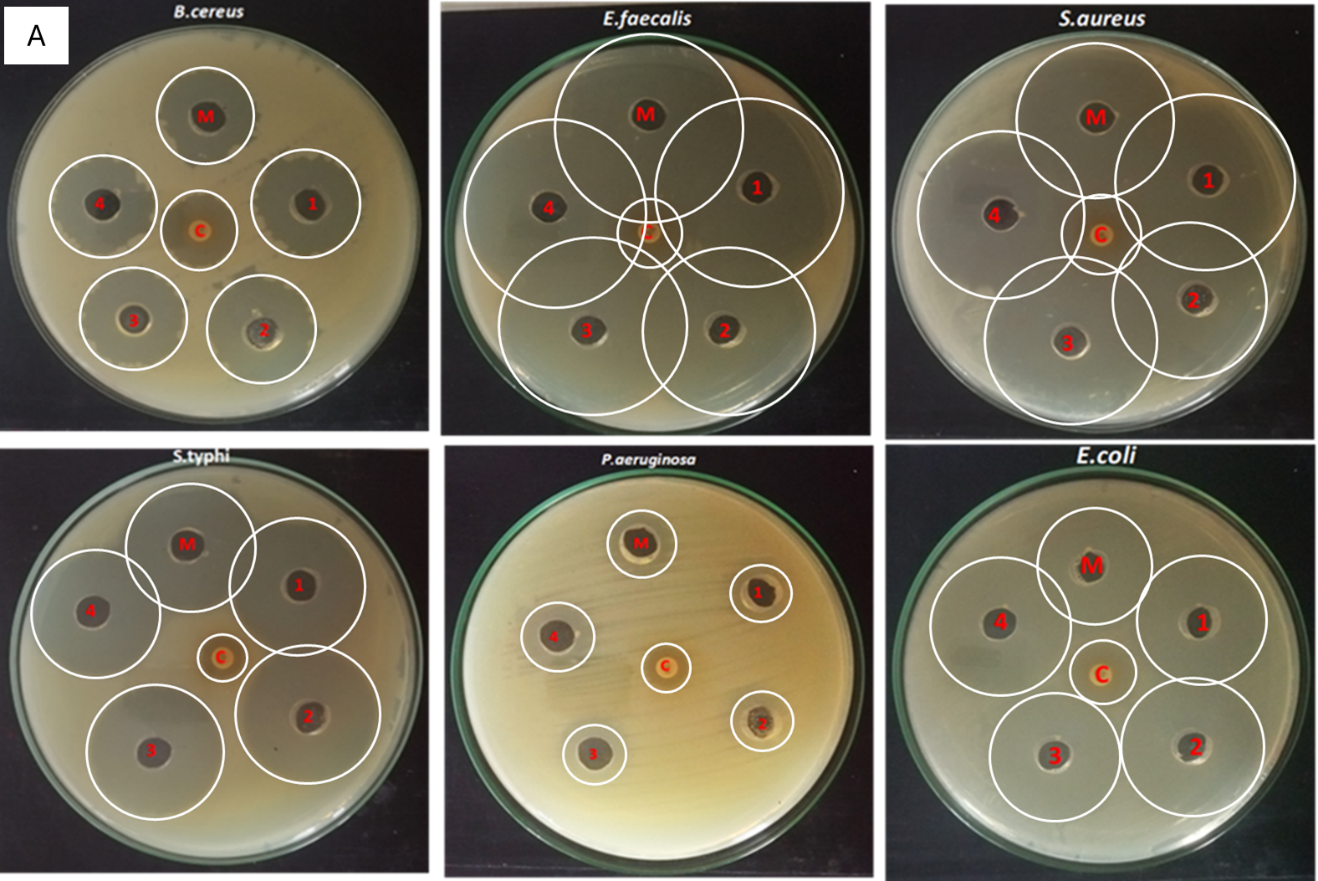


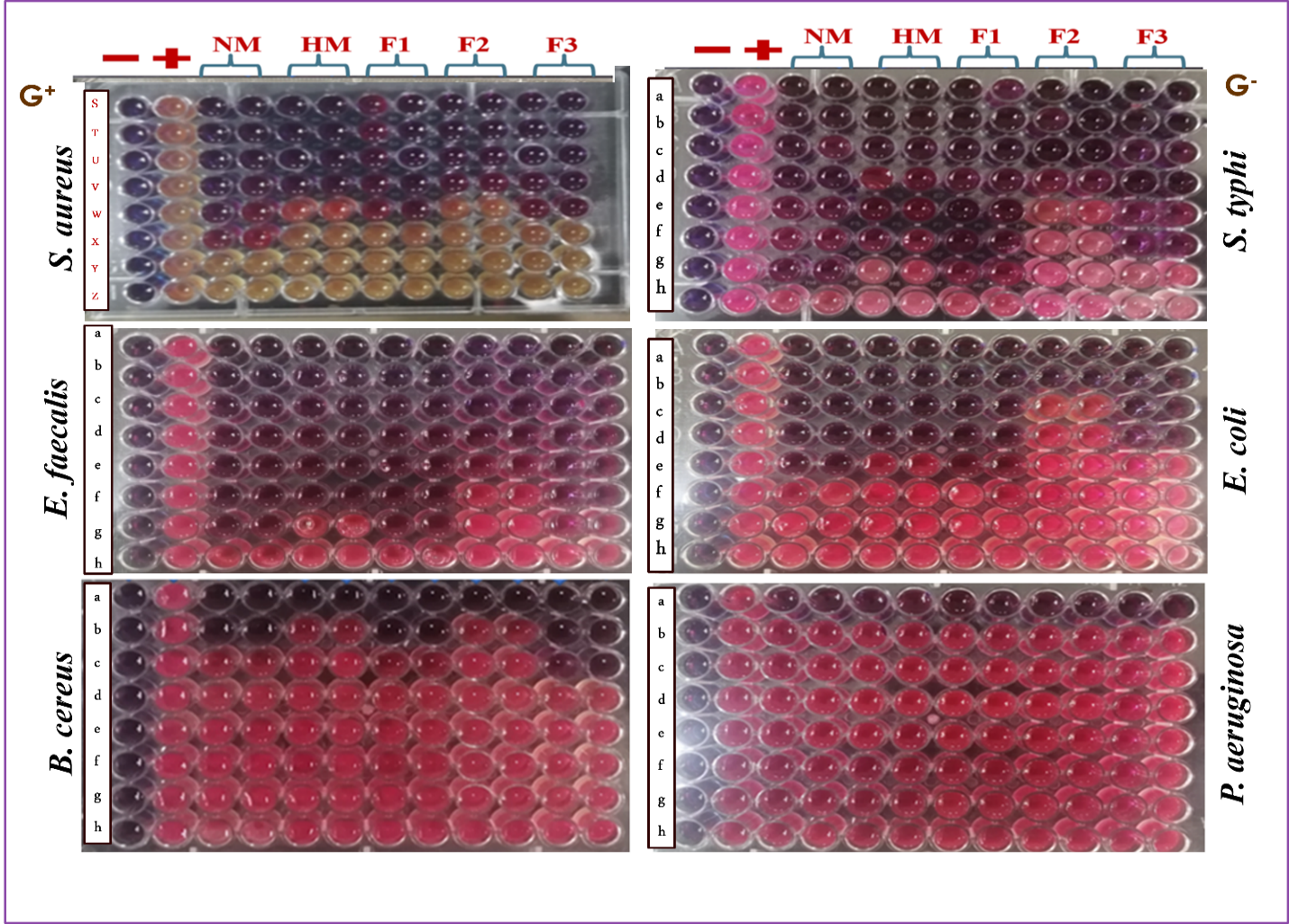


**B**

**Supplementary Figure 1.** Antibacterial activity of native (NM), hydrolysate (HM) and their alcalse hydrolysis fractions (F1, F2 and F3(.

**A**: (Agar well diffusion. HM). (1 Native melittin: NM as well as its hydrolysis fractions (2=F1, 3= F2, 4= F3, C = control).

**B**: Resazurin assay. The image color indicate the cellular survival after 24 h in Mueller Hinton (MH) broth [pink color indicates growth and blue means inhibition of growth; First columns (-ve) = Negative or sterility control (MH broth + sterile distilled water + indicator) without bacteria; second columns (+ve)= Positive Control ( MH broth + bacterial suspension + indicator ) without compounds. The letters (a,b,c,d,e,f,g,h) refer to peptide concentrations of 10000, 5000, 2500, 1250, 625. 312, 156, 78 µg/ml in each corresponding row, while letters S, T, U, V, W, X, Y, Z) refer to peptide concentrations of 78, 39, 19.5, 9.75, 4.87, 2.43, 1.21, 0.6 µg/ml.
